# Development of Novel ^18^F‑Labeled Selective Orexin‑2 Receptor Radioligands for Positron Emission Tomography

**DOI:** 10.1021/acsptsci.5c00474

**Published:** 2025-10-11

**Authors:** Jian Rong, Chunyu Zhao, Ahmad F. Chaudhary, Jiahui Chen, Yinlong Li, Xin Zhou, Zhendong Song, Zhenkun Sun, Yabiao Gao, Siyan Feng, Taoqian Zhao, Qi-Long Hu, Chongjiao Li, Jimmy Patel, Hongjie Yuan, Achi Haider, Steven H. Liang

**Affiliations:** † Department of Radiology and Imaging Sciences, 1371 Emory University, Atlanta, Georgia 30322, United States; ‡ Department of Pharmacology and Chemical Biology, 12239 Emory University School of Medicine, Atlanta, Georgia 30322, United States; § Department of Radiation Oncology, Emory University, Atlanta, Georgia 30322, United States

**Keywords:** orexin-2 receptor, OX_2_, [^18^F]OX_2_-2303, [^18^F]OX_2_-2304, PET

## Abstract

The orexin-2 receptor (OX_2_R), a G protein-coupled receptor activated by the neuropeptides, orexin A and B, plays an integral role in orchestrating motivation, feeding behavior, and the sleep-wake cycle. Pharmacological modulation of OX_2_R has shown therapeutic potential for a variety of central nervous system (CNS) diseases, most notably narcolepsy and insomnia. Noninvasive imaging of OX_2_R could enable the visualization of its regional distribution, facilitate assessments of target engagement, and support the development of OX_2_R-directed therapies. Nonetheless, there are currently no suitable radioligands available for imaging OX_2_R with positron emission tomography (PET). Herein, we report the design and evaluation of two novel PET ligand candidates, [^18^F]**1** ([^18^F]­OX_2_-2303) and [^18^F]**2** ([^18^F]­OX_2_-2304), as potential imaging probes for OX_2_R. Both candidates exhibit excellent OX_2_R binding affinity (*K*_i_ = 0.1 and 1 nM, respectively) and remarkable selectivity over OX_1_R (>600-fold). *In vitro* autoradiography confirmed robust and selective binding to OX_2_R in rat brain sections. *In vivo* PET imaging revealed low brain uptake at baseline, attributed to active efflux by P-glycoprotein (P-gp) and/or breast cancer resistance protein (BCRP). Furthermore, pharmacological inhibition of these efflux transporters markedly enhanced brain penetration and OX_2_R antagonists demonstrated notable blocking effects to OX_2_R tracers during these conditions. Collectively, [^18^F]**1** ([^18^F]­OX_2_-2303) and [^18^F]**2** ([^18^F]­OX_**2**_ **-**2304) constitute promising chemical starting points for the development of OX_2_R PET radioligands, although further medicinal chemistry optimization will be required to overcome transporter-mediated efflux from the brain.

## Graphical abstract

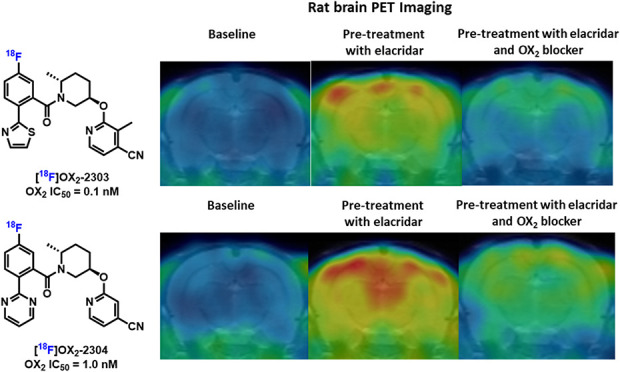

The orexin network mainly relies on two G protein-coupled receptors, the orexin-1 receptor (OX_1_R) and the orexin-2 receptor (OX_2_R), and their endogenous ligands, orexin-A and orexin-B.^1^ This neuromodulatory pathway exerts widespread influence across essential physiological domains, including the regulation of the sleep-wake cycle, feeding behavior, energy homeostasis, reward system, cognition, and mood.^2–7^ While orexin A exhibits comparable affinity for both receptor subtypes, orexin B shows marked selectivity for OX_2_R (>10-fold). Over the past decade, therapeutic interest in targeting the orexin system has grown, driven in part by the clinical success of dual orexin receptor antagonists (DORAs) and selective OX_2_R antagonists (SORAs) for the treatment of insomnia. Small molecule agents such as suvorexant, lemborexant, daridorexant, and seltorexant exemplify this strategy and have demonstrated clinical efficacy in modulating sleep physiology.^8–10^ Nonetheless, the precise contribution of OX_2_R to the pathophysiology of neuropsychiatric and neurodegenerative disorders remains not fully delineated. Molecular imaging offers a unique opportunity to elucidate the various roles of OX_2_R in both healthy and disease states, support target validation, and enable pharmacodynamic monitoring during early phase clinical development.

Positron emission tomography (PET) enables noninvasive, quantitative imaging of molecular targets within the living brain and has become an indispensable tool in translational neuroscience and drug development.^11–13^ To date, a series of OX_2_R PET radioligands have been reported, including [^11^C]­EMPA,^14^ [^11^C]­BBAC,^15^ [^11^C]­BBPC,^15^ [^11^C]­CW4,^16^ [^11^C]­FFMMCC,^17^ [^11^C]­MK-1064,^18^ [^18^F]­DAN-1,^19^ [^11^C]­CW24,^20^ [^18^F]­Seltorexant,^21^ [^11^C]­OX_2_-2201,^22^ [^11^C]­OX_2_-2202,^22^ [^11^C]­DMK-5220,^23^ [^11^C]­GSK1059865,^24^ and [^11^C]­ET1 ^24^ (Figure 1). However, these probes are plagued by suboptimal pharmacokinetics, including limited blood–brain barrier (BBB) permeability, poor binding selectivity, or rapid metabolic degradation, thus falling short of the criteria necessary for clinical translation. To address these challenges, we developed two ^18^F-labeled (half-life of fluorine-18: 110 min) small-molecule tracers, [^18^F]**1** ([^18^F]­OX_2_-2303) and [^18^F]**2** ([^18^F]­OX_2_-2304) derived from a chemotype previously shown to possess excellent OX_2_R affinity and selectivity. [^18^F]**1** and [^18^F]**2** were radiofluorinated and subsequently evaluated in a comprehensive series of *in vitro* and *in vivo* assays, including autoradiography, PET imaging, biodistribution, and metabolic stability studies. Our results position [^18^F]**1** and [^18^F]**2** as promising starting points for OX_2_R-targeted imaging, while identifying transporter efflux as a key barrier to optimal brain delivery *in vivo*.

**Figure 1:**
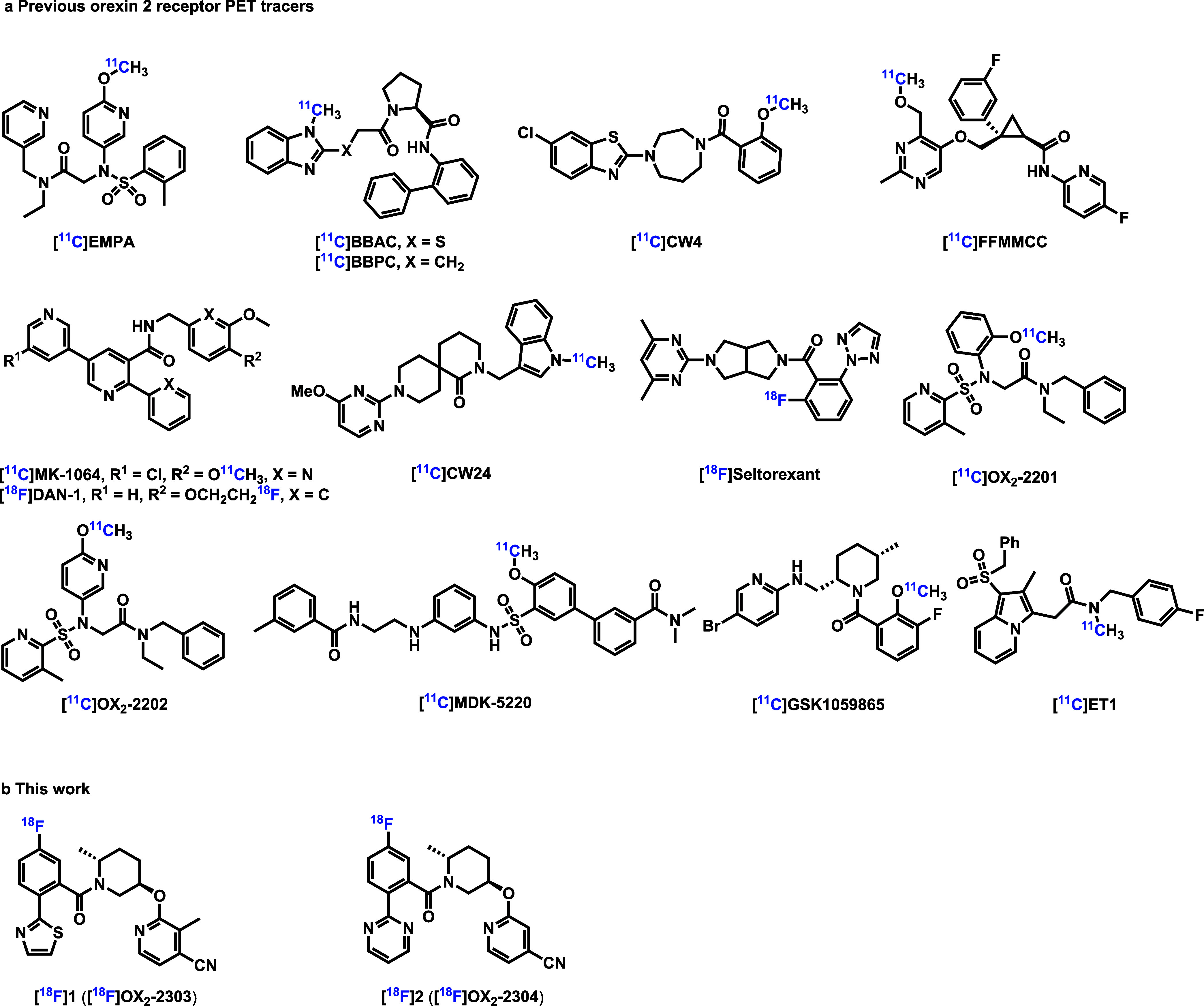
Representative OX_2_R PET radioligands. (a) Previous OX_2_R PET radioligands; (b) [^18^F]­OX_2_-2303 and [^18^F]­OX_2_-2304.

## Results and Discussion

To initiate the development of OX_2_R-selective PET tracers, we focused on two lead compounds, **1** and **2**, previously reported as antagonist candidates to possess subnanomolar-to-nanomolar binding affinity and excellent selectivity for OX_2_R over OX_1_R.^25,26^ As shown in Scheme 1, the synthesis of compound **1** was initiated from the hydrolysis of ester **3** under basic conditions to give acid **4** as a key intermediate in 97% yield. Subsequent condensation of **4** and 3-methyl-2-(((3*R*,6*R*)-6-methylpiperidin-3-yl)­oxy)­isonicotinonitrile afforded compound **1** in 33% yield. Compound **2** was prepared following a similar route, yielding intermediate **6** from ester **5** in 82%, followed by amide coupling with the corresponding amine to afford target compound **2** in 62% yield. Overall, the two-step syntheses yielded 31 and 51% of compounds **1** and **2**, respectively.

**Scheme 1:**
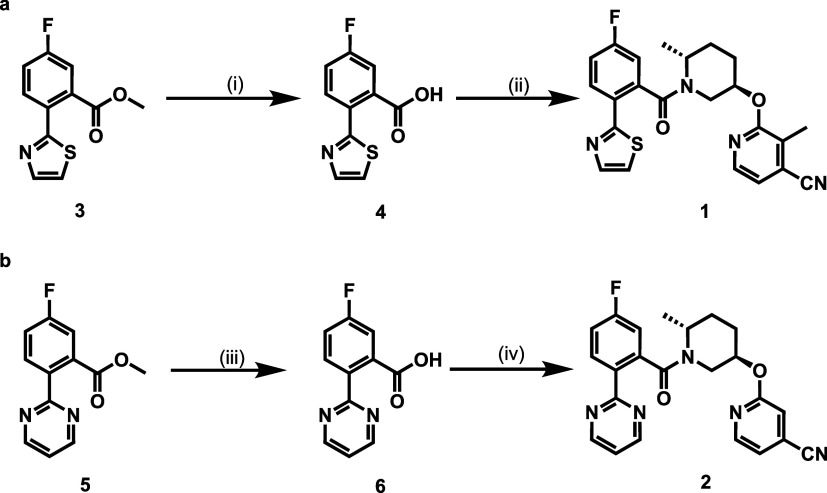
Preparation of Inhibitors **1** and **2** ^a^

a (a) Synthesis of 1, reaction conditions: (i) NaOH, MeOH, H_2_O, 25 °C, 2 h, 97% yield; (ii) 3-methyl-2-(((3*R*,6*R*)-6-methylpiperidin-3-yl)­oxy)­isonicotinonitrile trifluoroacetate, HATU, *N*,*N*-diisopropylethylamine, DMF, 25 °C, 2 h, 33% yield; (b) Synthesis of **2**, reaction conditions: (iii) NaOH, MeOH, H_2_O, 25 °C, 3 h, 82% yield; (iv) 2-(((3*R*,6*R*)-6-methylpiperidin-3-yl)­oxy)­isonicotinonitrile trifluoroacetate, HOBt, EDC·HCl, *N*,*N*-diisopropylethylamine, CH_2_Cl_2_, 25 °C, 6 h, 62% yield. HATU = hexafluorophosphate azabenzotriazole tetramethyl uronium, DMF = dimethylformamide, HOBt = hydroxybenzotriazole, EDC·HCl = 1-ethyl-3-(3-dimethylaminopropyl)­carbodiimide hydrochloride.

Both ligands exhibited excellent binding affinity toward OX_2_R, with *K*_i_ values of 0.1 nM for compound **1** and 1.0 nM for compound **2**, respectively (Table 1). Target selectivity over OX_1_R exceeded 600-fold for both compounds, satisfying a key prerequisite for selective OX_2_R imaging. Physicochemical profiling revealed topological polar surface areas (tPSA) and experimental log *D* values consistent with passive CNS permeability. CNS penetration was further predicted using Percepta, yielding log BB values of −0.14 and −0.36 for compounds **1** and **2**, respectively, well above the empirical threshold for BBB permeability (log BB > −1). Additionally, off-target binding screening was conducted for compounds **1** and **2** toward 59 major CNS targets (Figures S1 and S2). No off-target activity was observed, with the exception of low-affinity binding to sigma-2 (*K*_i_ = 2681 and 4728 nM for compounds **1** and **2**, respectively) and NR2B receptors (*K*_i_ = 9475 nM for compound **1**).

**Table 1:** Representative Pharmacological and Physicochemical Properties of **1** and **2**

pharmacology and physicochemical properties	compound 1	compound 2
*K*_i_/OX_2_ (nM)	0.1	1.0
*K*_i_/OX_1_ (nM)	89	658
selectivity (OX_2_R/OX_1_R)	890	658
MW (g/mol)	437	417
TPSA (Å^2^)	78.05	90.41
Log *D*	3.43	2.54
Log BB	–0.14	–0.36

As compounds **1** and **2** demonstrate excellent pharmacological and physicochemical properties, both compounds were labeled with fluorine-18 and further evaluated as potential PET ligands. As shown in Figures 2 and 3, [^18^F]**1** and [^18^F]**2** were synthesized via copper-mediated ^18^F-fluorination of borate precursors with [^18^F]­Et_4_NF in DMAc/*n*BuOH. [^18^F]**1** and [^18^F]**2** were obtained in 24% and 53% decay-corrected radiochemical yields (RCYs) with molar activities of 53 GBq/μmol and 38 GBq/μmol, respectively. Both probes showed radiochemical purities over 99%. Furthermore, *in vitro* stability tests of [^18^F]**1** and [^18^F]**2** were conducted in saline, serums, and liver microsomes across species. No appreciable radiometabolites were detected in saline, serum (mouse, rat, nonhuman primate, and human), and liver microsomes over the studied time intervals (Figures 2, 3, S3, and S4), indicating favorable tracer integrity for *in vitro* studies.

**Figure 2:**
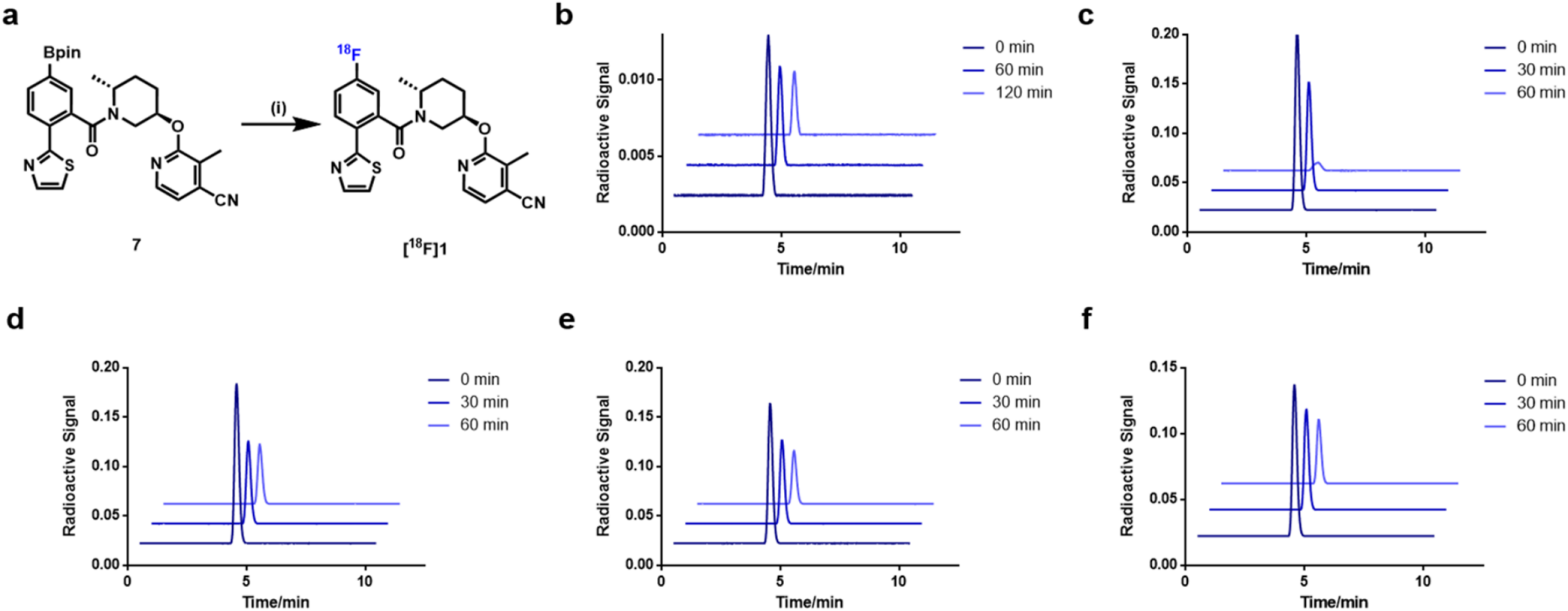
Radiosynthesis of [^18^F]**1** and stability tests of [^18^F]**1**. (a) Radiosynthesis of [^18^F]**1**, reaction conditions: (i) [Cu­(OTf)_2_(Py)_4_], [^18^F]­Et_4_NF, DMAc/*n*BuOH, 110 °C, 20 min, 24% RCY; (b) stability of [^18^F]**1** in saline; (c) stability of [^18^F]**1** in mouse serum; (d) stability of [^18^F]**1** in rat serum; (e) stability of [^18^F]**1** in NHP serum; (f) stability of [^18^F]**1** in human serum. DMAc = dimethylacetamide.

**Figure 3:**
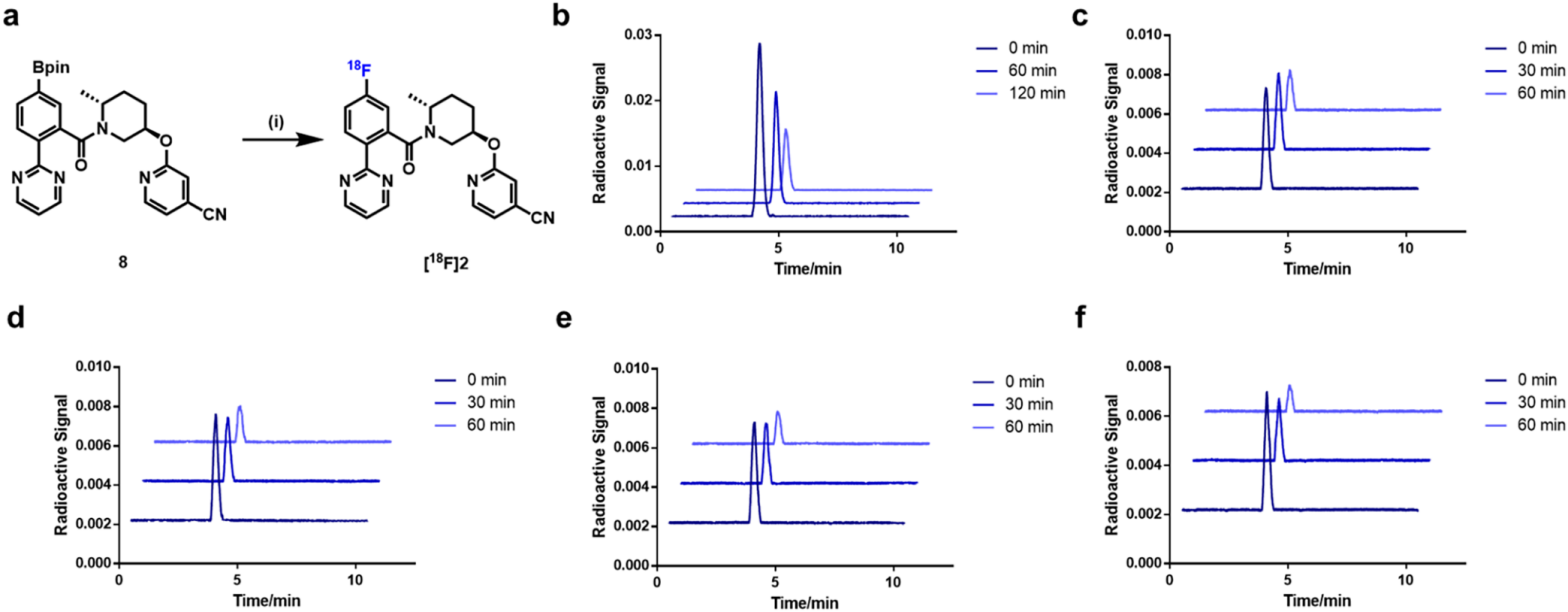
Radiosynthesis of [^18^F]**2** and tracer stability tests. (a) Radiosynthesis of [^18^F]**2**, reaction conditions: (i) [Cu­(OTf)_2_(Py)_4_], [^18^F]­Et_4_NF, DMAc/*n*BuOH, 110 °C, 20 min, 53% RCY; (b) stability of [^18^F]**2** in saline; (c) stability of [^18^F]**2** in mouse serum; (d) stability of [^18^F]**2** in rat serum; (e) stability of [^18^F]**2** in NHP serum; (f) stability of [^18^F]**2** in human serum. DMAc = dimethylacetamide.

To assess binding specificity in brain tissue, *in vitro* autoradiography was performed on rat brain sections using [^18^F]**1** and [^18^F]**2**. Both probes exhibited a regionally heterogeneous binding pattern, with prominent uptake in OX_2_R-enriched regions such as the cortex and hypothalamus (Figure 4).^27^ Blocking studies with nonradioactive OX_2_R ligands, EMPA, LSN2424100, or the authentic reference compounds resulted in a marked reduction in signal intensity across all regions, yielding signal reductions of 54–84% for [^18^F]**1** and 51–71% for [^18^F]**2**, respectively (Figure 4). These findings highlight the binding specificity of both tracers for OX_2_R *in vitro*.

**Figure 4:**
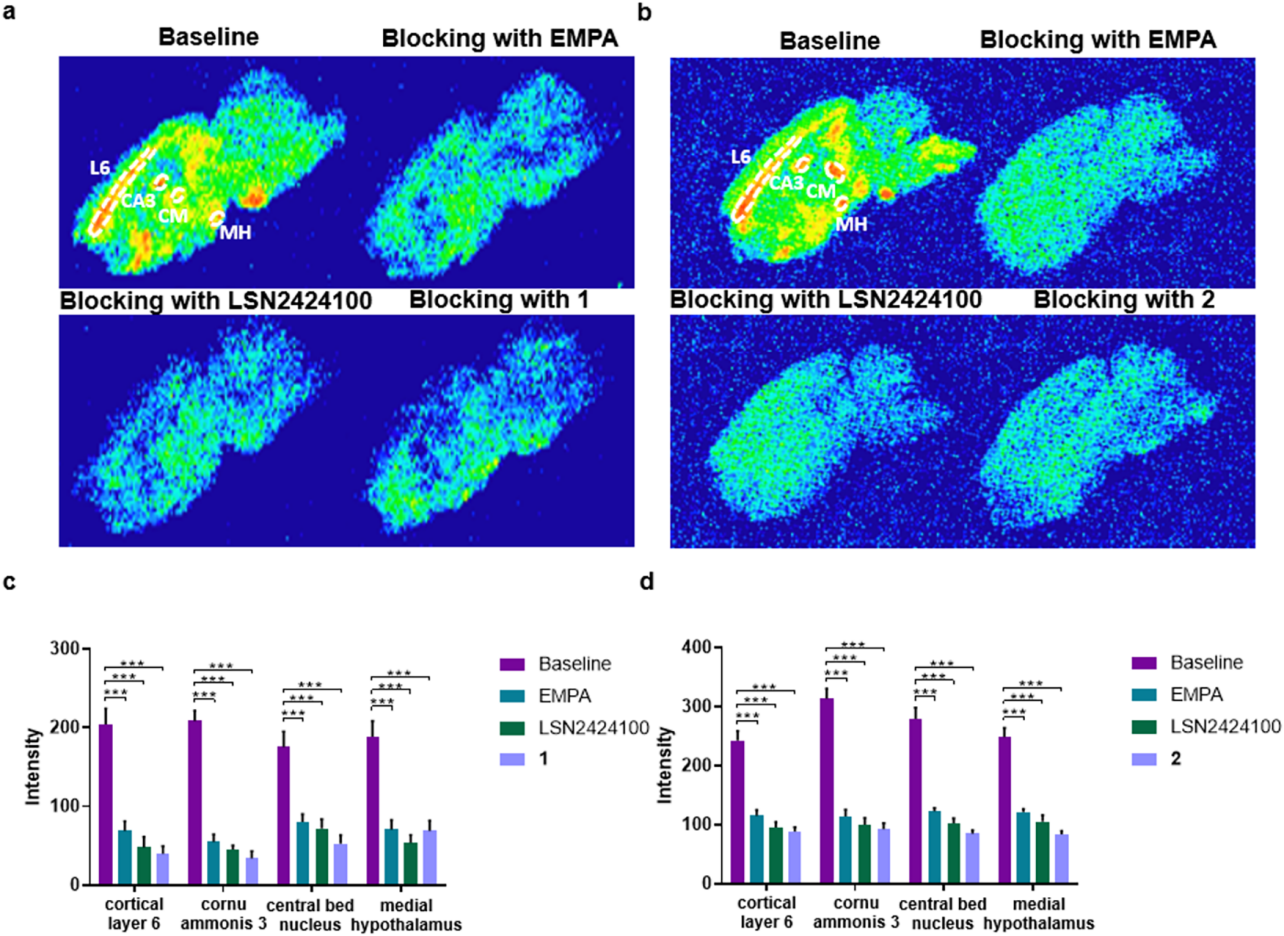
*In vitro* autoradiography with [^18^F]**1** and [^18^F]**2**. (a) Representative images of baseline and blocking (EMPA, LSN2424100, or **1**, 10 μM) studies of [^18^F]**1**; (b) representative images of baseline and blocking (EMPA, LSN2424100, or **2**, 10 μM) studies of [^18^F]**2**; (c) quantification of autoradiography of [^18^F]**1**; (d) quantification of autoradiography of [^18^F]**2**. L6 = cortical layer 6, CA3 = cornu ammonis 3, CM = central bed nucleus, MH = medial hypothalamus. All data are mean ± SD, *n* ≥ 6. Statistical analysis was calculated by one-way analysis of variance (ANOVA) test (***p ≤ 0.001).

Next, dynamic PET imaging studies were conducted in Sprague–Dawley rats to evaluate tracer performance *in vivo* (Figures 5 and 6). Under baseline conditions, both [^18^F]**1** and [^18^F]**2** reached peak SUVs of 2.1 and 1.8, respectively, at 0.5 min postinjection. However, both probes exhibited rapid washout, resulting in overall low brain retention (Figures S5 and S6). Given these observations, we hypothesized that active efflux transporters might restrict tracer brain exposure. Co-administration of elacridar, a dual P-gp/BCRP inhibitor, led to a significantly increased brain uptake, with >100% increase in SUV_0–60 min_ values for both tracers across brain regions. To evaluate *in vivo* binding specificity under efflux-inhibited conditions, additional blocking studies were performed using EMPA or LSN2424100 (1 mg/kg) following elacridar pretreatment. Both [^18^F]**1** and [^18^F]**2** exhibited a marked reduction in tracer uptake across all brain regions, with uptake reductions ranging from 40–43% for [^18^F]**1** and 23–46% for [^18^F]**2**. These results corroborate the *in vivo* specificity of both tracers for OX_2_R and implicate P-gp and/or BCRP efflux as key barriers limiting their baseline brain penetration.

**Figure 5:**
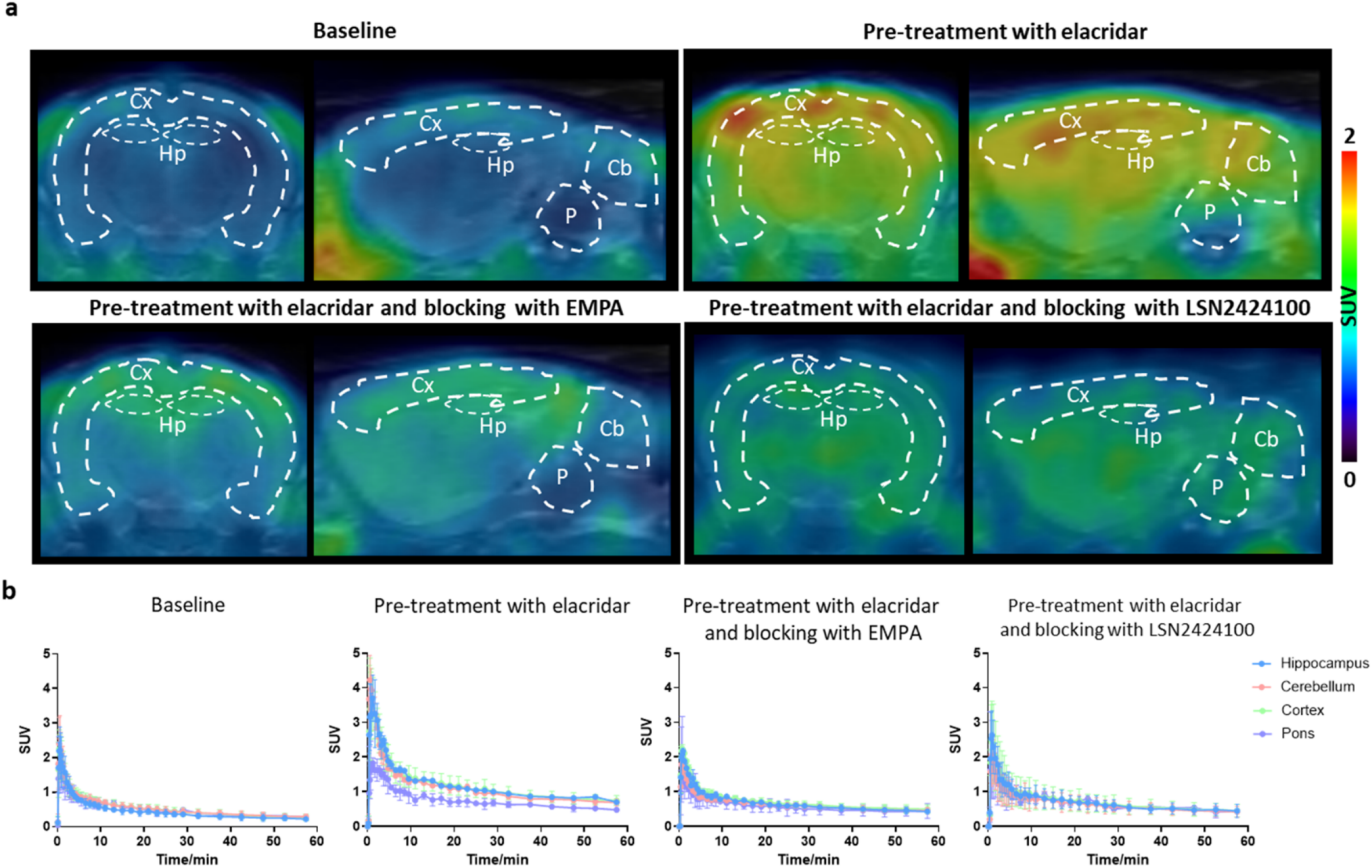
PET imaging with [^18^F]**1** in the rat brain. (a) Representative averaged images (0–60 min) of [^18^F]**1** in rat brains; (b) TACs of [^18^F]**1** in the hippocampus (Hp), cerebellum (Cb), cortex (Cx), and pons (P). All data are mean ± SD, *n* ≥ 3.

**Figure 6:**
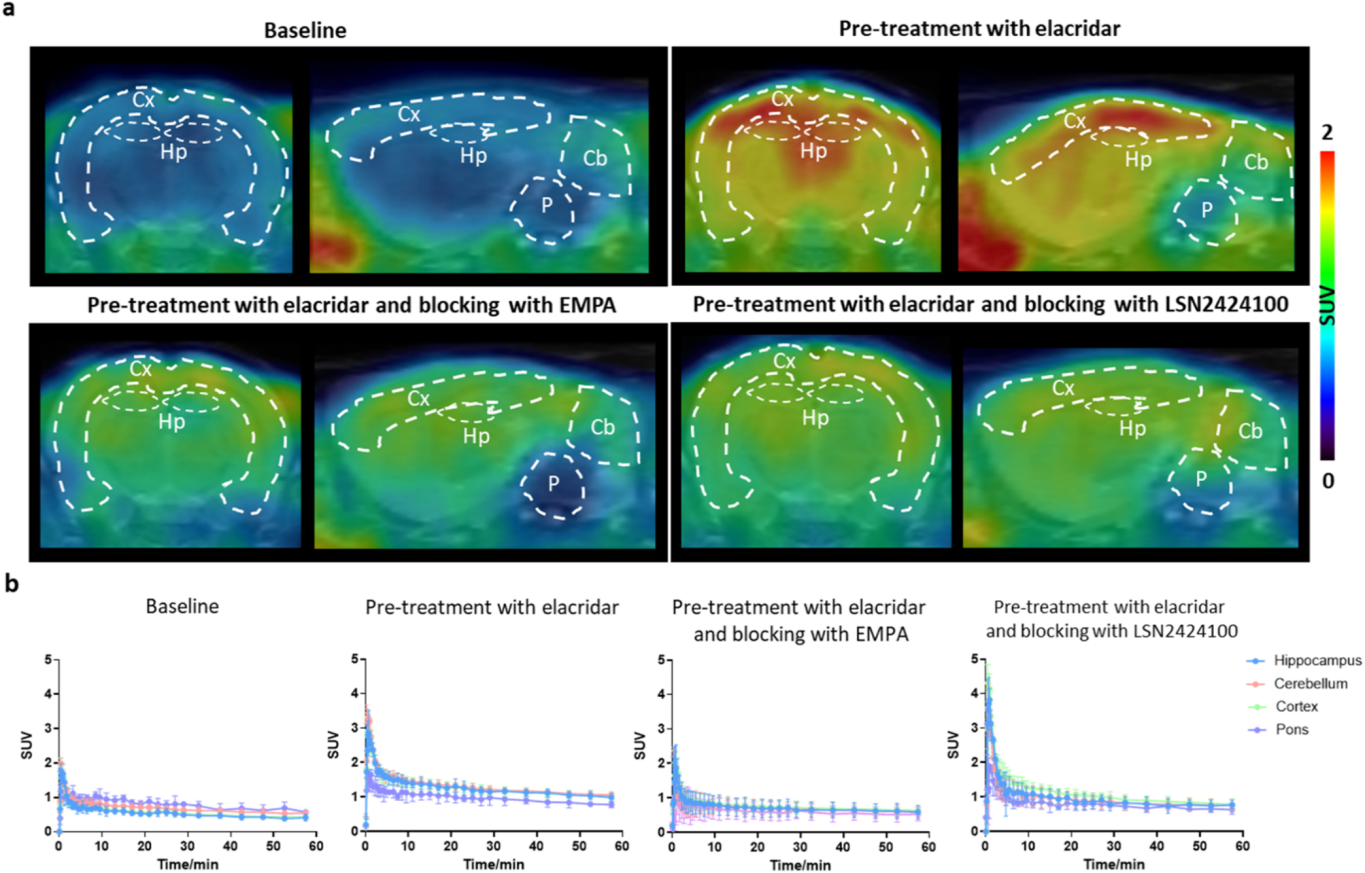
PET imaging of [^18^F]**2** in the rat brain. (a) Representative averaged images (0–60 min) of [^18^F]**2**; (b) TACs of [^18^F]**2** in the hippocampus (Hp), cerebellum (Cb), cortex (Cx), and pons (P). All data are mean ± SD, *n* ≥ 3.

To complement brain imaging studies, *ex vivo* biodistribution was assessed in CD-1 mice at 5, 15, 30, and 60 min postinjection (Figure 7). At early time points, tracer accumulation was greatest in the pancreas, kidney, liver, and small intestine (>5% ID/g), consistent with rapid systemic clearance via the hepatobiliary route. Brain uptake remained low throughout, whereas virtually no bone accumulation was observed at all measured time points, suggesting negligible *in vivo* defluorination.

**Figure 7:**
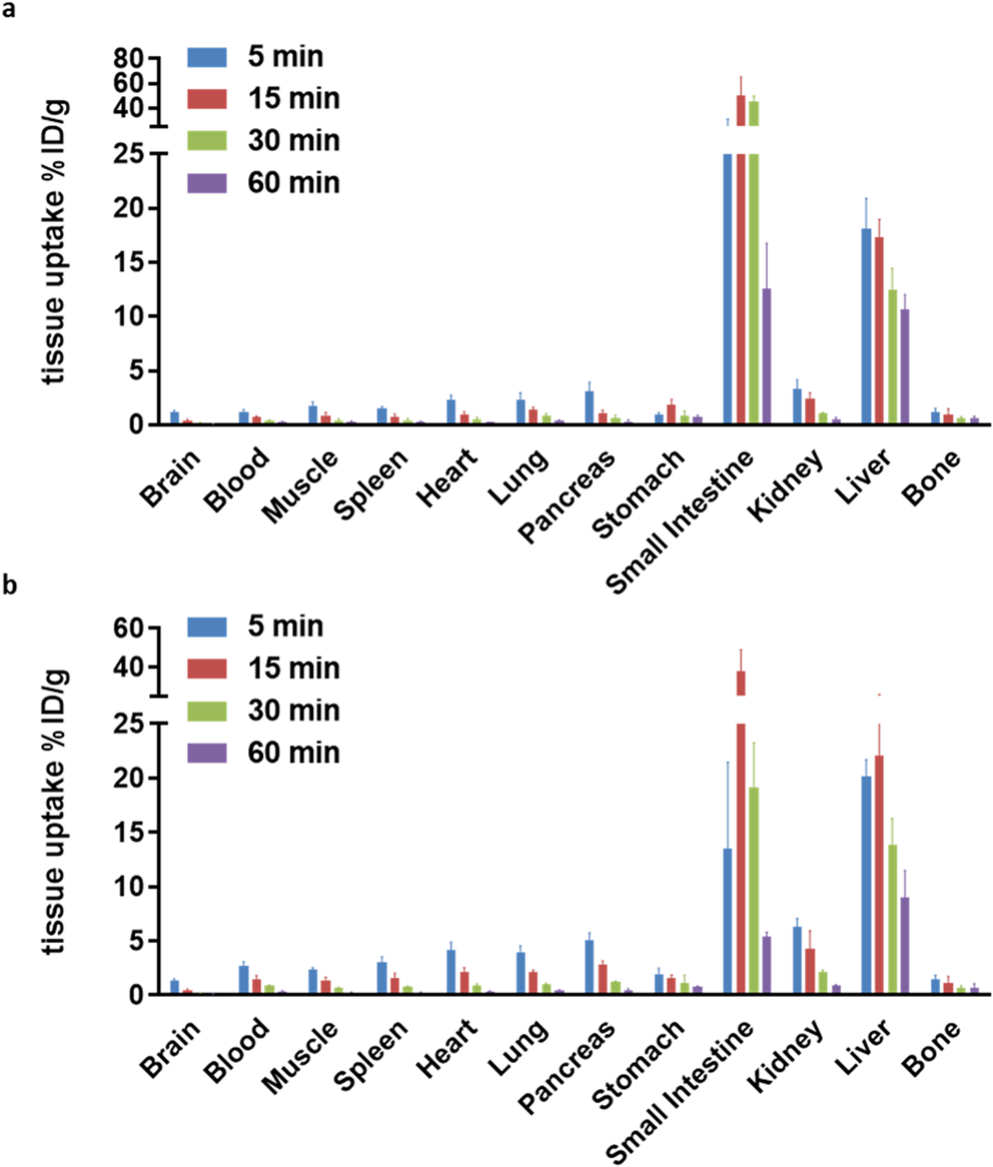
Whole-body biodistribution studies of [^18^F]**1** (a) and [^18^F]**2** (b) in mice. All data are mean ± SD, *n* = 4.

Metabolic stability was further assessed in rat plasma and brain homogenates at 30 min postinjection (Figure 8). High levels of intact parent tracer were detected in brain samples (ca. 90% for both tracers), while moderate degradation was observed in plasma (56% for [^18^F]**1** and 62% for [^18^F]**2**), suggesting favorable *in vivo* stability.

**Figure 8:**
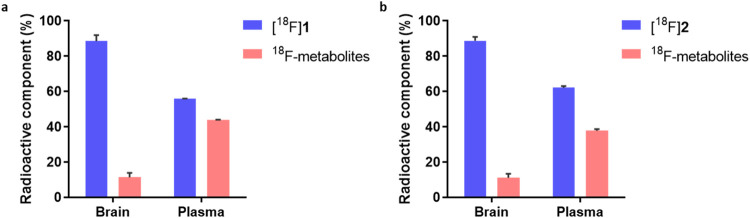
Radiometabolic analysis of [^18^F]**1** (a) and [^18^F]**2** (b). All data are mean ± SD, *n* = 3.

## Conclusions

We report the development and comprehensive preclinical evaluation of two novel ^18^F-labeled PET tracers, [^18^F]­OX2–2303 ([^18^F]**1**) and [^18^F]­OX2–2304 ([^18^F]**2**), designed to enable noninvasive imaging of the orexin-2 receptor (OX_2_R). Both tracers originate from chemotypes exhibiting excellent binding affinity (*K*_i_ = 0.1 and 1.0 nM, respectively) and remarkable selectivity over OX_1_R (>600-fold), and were synthesized in high radiochemical yields with excellent purity and molar activity. *In vitro* autoradiography confirmed the specific binding to OX_2_R in rat brain tissues, while PET imaging studies revealed limited brain penetration under baseline conditions, likely mediated by active efflux via P-gp and/or BCRP. Notably, coadministration of elacridar substantially improved tracer uptake and enabled the visualization of specific OX_2_R binding *in vivo*. These findings establish [^18^F]­OX2–2303 and [^18^F]­OX2–2304 as promising lead structures for OX_2_R PET imaging. While their target specificity and *in vivo* stability were suitable for PET imaging, future optimization efforts should focus on mitigating transporter-mediated efflux to enhance baseline brain exposure. Such refinements would pave the way for the application of OX_2_R imaging in patient stratification, target engagement studies, and the clinical development of orexin-based therapeutics.

## Experimental Sections

### Chemistry

#### 2-(((3*R*,6*R*)-1-(5-Fluoro-2-(thiazol-2-yl)­benzoyl)-6-methylpiperidin-3-yl)­oxy)-3-methylisonicotinonitrile (**1**)

To a solution of methyl 5-fluoro-2-(thiazol-2-yl) benzoate **3** (70 mg, 0.3 mmol, 1 equiv) in MeOH (1 mL), NaOH (23.6 mg, 0.59 mmol, 2 equiv) in H_2_O (0.5 mL) was added. The reaction was stirred at room temperature for 2 h. Then the pH of the mixture was adjusted to 6–7 with HCl (1 M, aq.). The mixture was diluted with water and ethyl acetate. The organic layer was dried over Na_2_SO_4_ and concentrated to give crude **4** (yellow solid, 65 mg, 97% yield).

To a solution of **4** (50 mg, 0.22 mmol, 1 equiv) and 3-methyl-2-(((3*R*,6*R*)-6-methylpiperidin-3-yl)­oxy)­isonicotinonitrile trifluoroacetate (72.5 mg, 0.22 mmol, 1 equiv) in DMF (1 mL), HATU (102.2 mg, 0.27 mmol, 1.2 equiv) and diisopropylethylamine (115.8 mg, 0.9 mmol, 4 equiv) were added. The reaction was stirred at room temperature for 2 h. The mixture was diluted with water and ethyl acetate. The organic layer was dried over Na_2_SO_4_, concentrated, purified by column chromatography, and recrystallized in MeCN to give **1** (white solid, 32 mg, 33% yield, two rotamers). ^1^H NMR (400 MHz, CDCl_3_): δ 8.10–7.69 (m, 3H), 7.40 [7.37] (d, *J* = 3.2 Hz, 1H), 7.25–6.56 (m, 3H), 5.42–4.98 (m, 2H), 3.84–3.66 (m, 1H), 3.17 [3.06] (d, *J* = 14.8 Hz, 1H), 2.49 (s, 3H), 2.28–2.19 (m, 1H), 1.99–1.97 (m, 2H), 1.53 (d, *J* = 14.0 Hz, 1H), 1.29 (d, *J* = 6.8 Hz, 3H). ^19^F NMR (382 MHz, CDCl_3_): δ −109.6 (m, 1F). ^13^C NMR (100 MHz, CDCl_3_): δ 168.91, 164.55, 162.76 (d, *J* = 251.3 Hz), 161.12, 144.52, 143.60, 137.70 (d, *J* = 7.2 Hz), 130.93 (d, *J* = 8.1 Hz), 126.73, 124.83, 122.51, 120.05, 118.13, 115.99, 115.92 (d, *J* = 21.6 Hz), 114.52 (d, *J* = 23.1 Hz), 68.81, 44.05, 43.80, 24.82, 23.57, 14.43, 13.81. LRMS (ESI): C_23_H_22_FN_4_O_2_^+^ (M + H^+^): 437.1, found: 437.2. HRMS (ESI): exact mass calcd for C_23_H_22_FN_4_O_2_^+^ (M + H^+^): 437.1442, found: 437.1458.

#### 2-(((3*R*,6*R*)-1-(5-Fluoro-2-(pyrimidin-2-yl)­benzoyl)-6-methylpiperidin-3-yl)­oxy)­isonicotinonitrile (**2**)

Through a similar procedure described for **4**, compound **6** was obtained (white solid, 50 mg, 82% yield).

To a solution of **6** (43 mg, 0.2 mmol, 1.4 equiv) and 2-(((3*R*,6*R*)-6-methylpiperidin-3-yl)­oxy)­isonicotinonitrile trifluoroacetate (42.8 mg, 0.14 mmol, 1 equiv) in CH_2_Cl_2_ (1 mL) was added *N*,*N*-diisopropylethylamine (102 mg, 0.79 mmol, 5.6 equiv), hydroxybenzotriazole (HOBt, 32 mg, 0.24 mmol, 1.7 equiv) and EDC·HCl (45.8 mg, 0.24 mmol, 1.7 equiv). The reaction was stirred at 25 °C for 6 h. Then the solution was concentrated and purified by column chromatography and reverse HPLC to afford 2-(((3*R*, 6*R*)-1-(5-fluoro-2-(pyrimidin-2-yl) benzoyl)-6-methylpiperidin-3-yl)­oxy)­isonicotinonitrile **2** (white solid, 36 mg, 62% yield, two rotamers). ^1^H NMR (400 MHz, CDCl_3_) δ 8.84 [8.71] (d, *J* = 4.8 Hz, 2H), 8.39 [8.29] (dd, *J* = 8.8, 5.6 Hz, 1H), 7.98 (d, *J* = 4.8 Hz, 1H), 7.17 (t, *J* = 4.8 Hz, 1H), 7.05–6.64 (m, 4H), 5.38–4.99 (m, 2H), 3.93 [3.72] (d, *J* = 14.4 Hz, 1H), 3.20 [3.14] (d, *J* = 15.2 Hz, 1H), 2.33–2.20 (m, 1H), 2.00 (m, 2H), 1.54 (d, *J* = 13.2 Hz, 1H), 1.36 (d, *J* = 6.8 Hz, 3H). ^19^F NMR (382 MHz, CDCl_3_): δ −108.9 (m, 1F). ^13^C NMR (100 MHz, CDCl_3_): δ 170.48, 163.55 (d, *J* = 251.4 Hz), 163.35, 163.05, 156.97, 148.10, 139.70 (d, *J* = 7.2 Hz), 132.01 (d, *J* = 8.6 Hz), 130.97, 123.09, 119.23, 117.88, 116.44, 115.48 (d, *J* = 21.2 Hz), 114.56 (d, *J* = 23.1 Hz), 114.47, 69.04, 43.99, 43.73, 24.62, 23.75, 13.91. LRMS (ESI): C_23_H_21_FN_5_O_2_^+^ (M + H^+^): 418.2, found: 418.2. HRMS (ESI): exact mass calcd for C_23_H_21_FN_5_O_2_^+^ (M + H^+^): 418.1674, found: 418.1686.

#### 3-Methyl-2-(((3*R*,6*R*)-6-methyl-1-(5-(4,4,5,5-tetramethyl-1,3,2-dioxaborolan-2-yl)-2-(thiazol-2-yl)­benzoyl)­piperidin-3-yl)­oxy)­isonicotinonitrile (**7**)

To a solution of 2-(((3*R*,6*R*)-1-(5-bromo-2-(thiazol-2-yl)­benzoyl)-6-methylpiperidin-3-yl)­oxy)-3-methylisonicotinonitrile (170 mg, 0.34 mmol, 1 equiv) in *t*BuOH (2 mL), KOAc (100.6 mg, 1.03 mmol, 3 equiv), B_2_Pin_2_ (433.9 mg, 1.71 mmol, 5 equiv) and Xphos-Pd-G2 (27 mg, 0.034 mmol, 0.1 equiv) were added. The reaction was stirred at 90 °C for 4 h under N_2_ atmosphere. Then the mixture was diluted with water and ethyl acetate. The organic layer was dried over Na_2_SO_4_ and concentrated to give a crude pruduct.

The crude product was dissolved in CH_2_Cl_2_ (5 mL), and then pinacol (401 mg, 3.4 mmol, 10 equiv) and MgSO_4_ (120 mg, 1.7 mmol, 5 equiv) were added. The reaction was stirred at room temperature for 16 h. The reaction mixture was filtered, and the filtrate was diluted with water and ethyl acetate. The organic layer was dried over Na_2_SO_4_ and purified by column chromatography to afford **7** (white solid, 45 mg, 24%, two rotamers). ^1^H NMR (400 MHz, CDCl_3_) δ 8.10–7.73 (m, 5H), 7.41 [7.40] (d, *J* = 3.2 Hz, 1H), 7.03 [6.94] (d, *J* = 5.2 Hz, 1H), 5.42–5.05 (m, 2H), 3.86–3.65 (m, 1H), 3.25 [3.10] (d, *J* = 14.8 Hz, 1H), 2.59 [2.57] (s, 3H), 2.05–1.84 (m, 3H), 1.48 (d, *J* = 14.0 Hz, 1H), 1.36–1.30 (m, 15H). ^13^C NMR (150 MHz, CDCl_3_): δ 171.30, 166.23, 161.17, 144.53, 142.54, 135.40, 135.11, 134.30, 131.46, 128.45, 125.85, 121.99, 120.71, 118.09, 117.82, 116.24, 116.19, 84.50, 84.39, 68.63, 45.51, 43.74, 25.39, 24.71, 22.70, 21.21, 14.86, 13.96. HRMS (ESI): exact mass calcd for C_29_H_34_BN_4_O_4_S^+^ (M + H^+^): 545.2388, found: 545.2407.

#### 2-(((3*R*,6*R*)-6-Methyl-1-(2-(pyrimidin-2-yl)-5-(4,4,5,5-tetramethyl-1,3,2-dioxaborolan-2-yl)­benzoyl)­piperidin-3-yl)­oxy)­isonicotinonitrile (**8**)

To 2-(((3*R*,6*R*)-1-(5-bromo-2-(pyrimidin-2-yl)­benzoyl)-6-methylpiperidin-3-yl)­oxy)­isonicotinonitrile (160 mg, 0.33 mmol, 1 equiv) in *t-*butanol (3 mL), B_2_Pin_2_ (424.7 mg, 1.67 mmol, 5 equiv), KOAc (98 mg, 1 mmol, 3 equiv) and XPhos-Pd-G2 (26 mg, 0.033 mmol, 0.1 equiv) were added and the mixture was stirred at 90 °C for 3 h. Then the reaction was diluted with water and ethyl acetate, and the organic phase was dried over Na_2_SO_4_, concentrated, and purified by HPLC and column chromatography to afford **8** (off-white solid, 90 mg, 52% yield, two rotamers). ^1^H NMR (400 MHz, CDCl_3_) δ 8.78 [8.76] (d, *J* = 4.8 Hz, 2H), 8.33–7.84 (m, 4H), 7.22 (t, *J* = 4.8 Hz, 1H), 7.13–7.05 (m, 1H), 6.99 (d, *J* = 3.6 Hz, 1H), 5.40–5.01 (m, 2H), 3.94 [3.77] (d, *J* = 14.4 Hz, 1H), 3.31 [3.14] (d, *J* = 14.4 Hz, 1H), 2.18–1.93 (m, 3H), 1.49 (d, *J* = 13.6 Hz, 1H), 1.37–1.35 (m, 15H). ^13^C NMR (150 MHz, CDCl_3_): δ 172.00, 164.16, 163.26, 157.14, 156.97, 148.58, 148.18, 136.94, 136.82, 135.26, 135.10, 134.26, 128.94, 122.64, 119.49, 117.80, 117.60, 116.70, 114.75, 84.32, 83.65, 69.55, 45.40, 43.68, 25.28, 25.17, 24.90, 24.50, 13.96. HRMS (ESI): exact mass calcd for C_29_H_33_BN_5_O_4_^+^ (M + H^+^): 526.2620, found: 526.2636.

### Radiochemistry

[^18^F]­fluoride in water was added to a V-vial with tetraethylammonium bicarbonate in MeOH (1 mg/0.5 mL). The vial was dried with nitrogen gas at 110 °C. After that, precursor **7** or **8** (2 mg) and CuOTf_2_(Py)_4_ (7 mg) in DMAc/*n*BuOH (0.2/0.1 mL) was added to dried [^18^F]­Et_4_NF and the reaction was heated at 110 °C in the air for 20 min. Then the mixture was diluted with water and passed through a Sep-Pak C18 light cartridge, and the product was eluted with MeCN (1 mL), diluted with water, and purified with HPLC to afford [^18^F]**1** and [^18^F]**2**.

### *In Vitro* Autoradiography Study

According to a previous report,^28^ rat brain sections were incubated with Tris-HCl buffer (50 mM) and then with [^18^F]**1** or [^18^F]**2**. In the blocking study, brain sections were incubated with the tracer in the presence of **1**, **2**, EMPA, or LSN2424100 (10 μM). Then the brain sections were washed with the cold buffer, dried, exposed to an imaging screen, and scanned with a Typhoon biomolecular imager.

### PET Imaging Study

All animal studies were performed following the guidelines of institutional animal care and use committee of the Emory University (protocols #PROTO202200003 and PROTO202200076).

PET imaging was performed according to previous reports^29,30^ with minor modifications. [^18^F]**1** or [^18^F]**2** (40 μCi) was administered via the tail vein of SD rats, and dynamic scans were performed with a G8 PET scanner (Sofie) for 60 min. In the blocking study, elacridar (5 mg/kg) and OX_2_R antagonist (EMPA or LSN2424100, 1 mg/kg) were injected at 20 and 10 min before the administration of the tracer, respectively.

### Whole-Body *Ex Vivo* Biodistribution Study

According to a previous report,^31^ CD-1 mice were sacrificed at 5, 15, 30, and 60 min after the administration of [^18^F]**1** or [^18^F]**2** (10 μCi/100 μL) via the tail vein. Major organs of interest were collected, weighed, and measured by a γ counter.

### Radiometabolic Analysis

According to a previous report,^32^ SD rats were euthanized 30 min after the administration of [^18^F]**1** or [^18^F]**2**. The brain was collected, homogenized with cold MeCN and PBS, and centrifuged (14,000*g*, 5 min) at 4 °C. The supernatant was analyzed by HPLC, and both tracers and radiometabolites were collected and measured by a γ counter. The same procedure was conducted for plasma.

## Supplementary Material



## Notes

Off-target evaluation; stability tests; time-activity curves; whole-body biodistribution; mass calibration curves, HPLC radio-chromatograms, and ^1^H, ^19^F, and ^13^C NMR spectra (PDF)

## Abbreviations

BBB: blood–brain barrier
BCRP: breast cancer resistance protein
CNS: central nervous system
DORA: dual orexin receptor antagonist
OX_1_R: orexin-1 receptor
OX_2_R: orexin-2 receptor
PET: positron emission tomography
P-gp: P-glycoprotein
RCY: radiochemical yield
SORA: selective OX2R antagonist
tPSA: topological polar surface areas
