# Supplementary material for: Development of Novel 18F‑Labeled Selective Orexin‑2 Receptor Radioligands for Positron Emission Tomography
Source: ACS Pharmacol Transl Sci. 2025 Oct 11;8(11):4070–9. doi: 10.1021/acsptsci.5c00474 (PMC12624432; doi:10.1021/acsptsci.5c00474)

Supporting Information

Development of Novel ^{18}F-Labeled Selective Orexin-2 Receptor Radioligands for Positron Emission Tomography

Jian Rong[1], Chunyu Zhao[1], Ahmad F. Chaudhary[1], Jiahui Chen[1], Yinlong Li[1], Xin Zhou[1], Zhendong Song[1], Zhenkun Sun[2], Yabiao Gao[1], Siyan Feng[1], Taoqian Zhao[1], Qi-Long Hu[1], Chongjiao Li[1], Jimmy Patel,[1,3] Hongjie Yuan,[2] Achi Haider[1], and Steven H Liang[1,*]

[1]Department of Radiology and Imaging Sciences, Emory University, Atlanta, Georgia 30322, United States

[2]Department of Pharmacology and Chemical Biology, Emory University School of Medicine, Atlanta, Georgia 30322, United States

[3]Department of Radiation Oncology, Emory University, Atlanta, Georgia 30322, United States

*E-mail: steven.liang@emory.edu

Table of contents

1. **Supporting Figures and Tables**.....S2

Figure S1 Off-target pharmacological evaluation of compound **1**.....S3

Figure S2 Off-target pharmacological evaluation of compound **2**.....S3

Figure S3 Stability of [^{18}F]**1** in the mouse, rat, and human liver microsomes.....S4

Figure S4 Stability of [^{18}F]**2** in the mouse, rat, and human liver microsomes.....S4

Figure S5 TACs of [^{18}F]**1** in the whole rat brain.....S4

Figure S6 TACs of [^{18}F]**2** in the whole rat brain.....S5

Table S1 Whole-body biodistribution study of [^{18}F]**1** in CD-1 mice.....S5

Table S2 Whole-body biodistribution study of [^{18}F]**2** in CD-1 mice.....S6

2. Mass calibration curves of compounds 1 and 2.....S6

3. HPLC radio-chromatograms of [^{18}F]1 and [^{18}F]2.....S8

4. ^{1}H, ^{19}F, and ^{13}C NMR spectra of isolated compounds.....S14

1. Supporting Figures and Tables

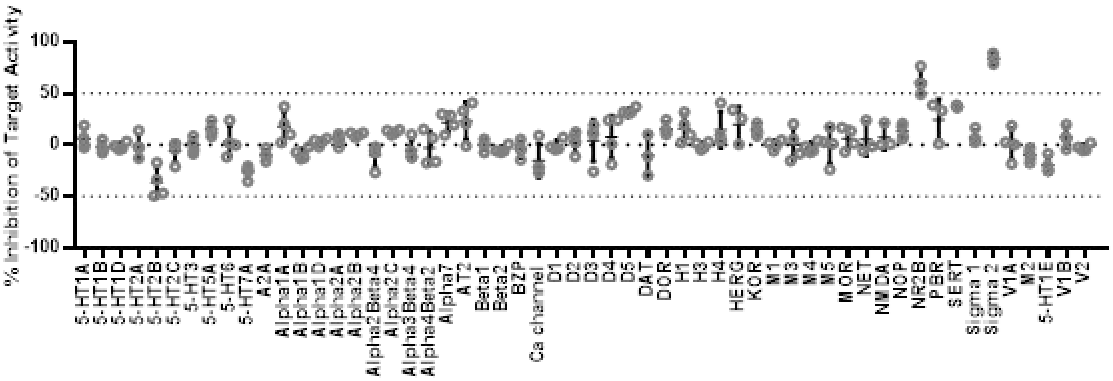

Figure S1. Off-target pharmacological evaluation of compound **1**. Initial screening of 59 major CNS targets at a concentration of **1** at 10 μM, including common GPCRs, enzymes, ion channels, and transporters. All data are mean ± SD (n ≥ 3). No significant off-target binding (> 50%) was observed, except NR2B (Ki = 9475 nM) and sigma 2 (Ki = 2681 nM).

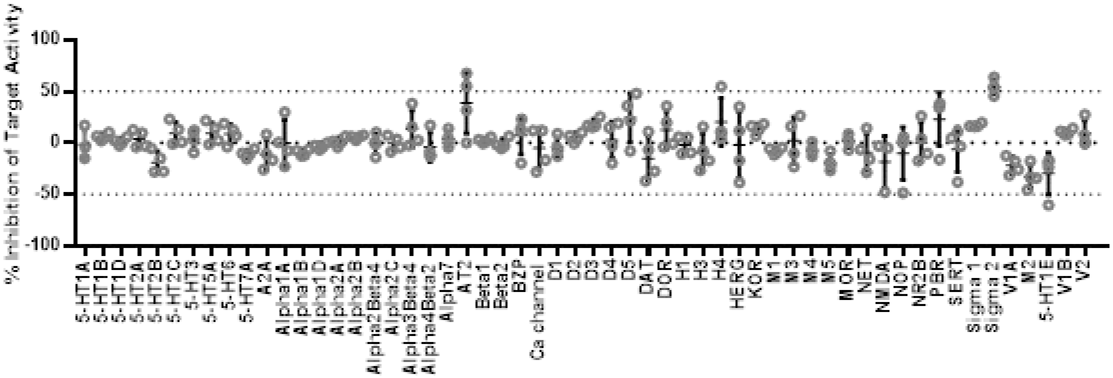

Figure S2. Off-target pharmacological evaluation of compound **2**. Initial screening of 59 major CNS targets at a concentration of **2** at 10 μM, including common GPCRs, enzymes, ion channels, and transporters. All data are mean ± SD (n ≥ 3). No significant off-target binding (> 50%) was observed, except sigma 2 (Ki = 4728 nM).

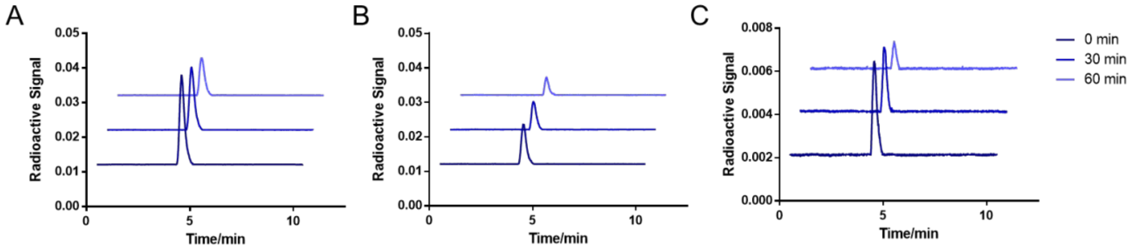

Figure S3. Stability of [^{18}F]**1** in liver microsomes. (A) Stability of [^{18}F]**1** in mouse liver microsome; (B) Stability of [^{18}F]**1** in rat liver microsome; (C) Stability of [^{18}F]**1** in human liver microsome.

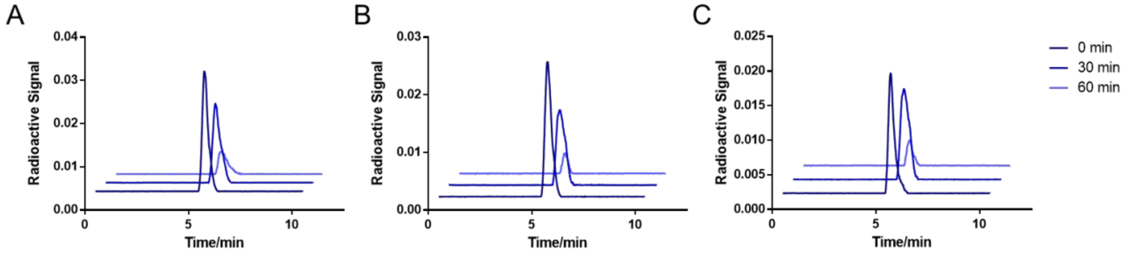

Figure S4. Stability of [^{18}F]**2** in liver microsomes. (A) Stability of [^{18}F]**2** in mouse liver microsome; (B) Stability of [^{18}F]**2** in rat liver microsome; (C) Stability of [^{18}F]**2** in human liver microsome.

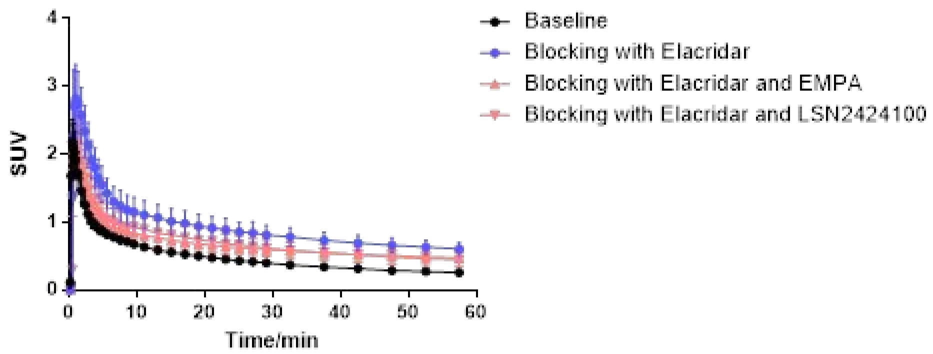

Figure S5. TACs of [^{18}F]**1** in the whole rat brain under baseline and blocking (elacridar, elacridar and EMPA, or elacridar and LSN2424100**;** elacridar in 5 mg/kg, EMPA and LSN2424100 in 1 mg/kg) conditions; All data are mean ± SD, n ≥ 3.

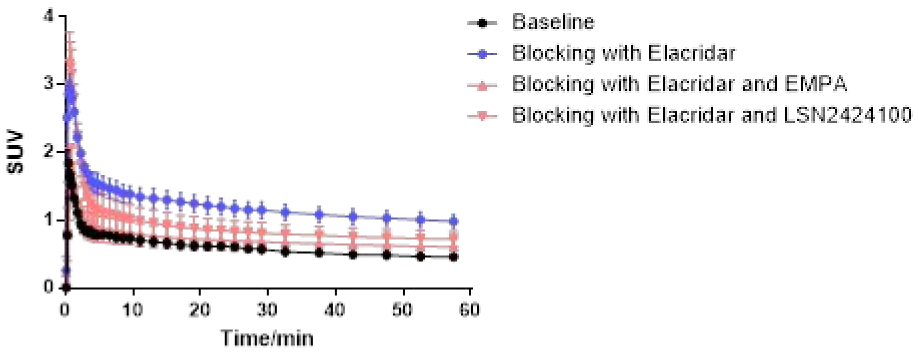

Figure S6. TACs of [^{18}F]**2** in the whole rat brain under baseline and blocking (elacridar, elacridar and EMPA, or elacridar and LSN2424100**;** elacridar in 5 mg/kg, EMPA and LSN2424100 in 1 mg/kg) conditions; All data are mean ± SD, n ≥ 3.

Table S1. Whole-body *ex vivo* biodistribution study of [^{18}F]**1** in CD-1 mice. All data are mean ± SD, n = 4.

	5 min			15 min			30 min			60 min		
%ID/g	Mean	SD	N	Mean	SD	N	Mean	SD	N	Mean	SD	N
A. Brain	1.10	0.20	**4**	0.41	0.09	**4**	0.17	0.05	**4**	0.06	0.00	**4**
B. Blood	1.20	0.18	**4**	0.66	0.13	**4**	0.38	0.05	**4**	0.27	0.02	**4**
C. Muscle	1.74	0.34	**4**	0.85	0.25	**4**	0.40	0.14	**4**	0.25	0.10	**4**
D. Spleen	1.44	0.24	**4**	0.76	0.22	**4**	0.41	0.17	**4**	0.20	0.13	**4**
E. Heart	2.23	0.48	**4**	0.94	0.26	**4**	0.51	0.17	**4**	0.23	0.01	**4**
F. Lung	2.33	0.61	**4**	1.42	0.20	**4**	0.78	0.25	**4**	0.41	0.01	**4**
G. Pancreas	3.09	0.81	**4**	1.09	0.24	**4**	0.60	0.25	**4**	0.30	0.13	**4**
H. Stomach	0.97	0.14	**4**	1.36	0.51	**4**	0.80	0.44	**4**	0.65	0.22	**4**
I. Small Intestir	25.52	5.66	**4**	50.35	15.00	**4**	44.87	4.99	**4**	12.53	4.24	**4**
J. Kidney	3.35	0.80	**4**	2.35	0.59	**4**	1.01	0.09	**4**	0.49	0.16	**4**
K. Liver	18.15	2.75	**4**	17.35	1.59	**4**	12.48	1.96	**4**	10.59	1.43	**4**
L. Bone	1.10	0.40	**4**	0.92	0.56	**4**	0.62	0.14	**4**	0.61	0.16	**4**

Table S2. Whole-body *ex vivo* biodistribution study of [^{18}F]**2** in CD-1 mice. All data are mean ± SD, n = 4.

	5 min			15 min			30 min			60 min		
%ID/g	Mean	SD	N	Mean	SD	N	Mean	SD	N	Mean	SD	N
A. Brain	1.31	0.20	4	0.49	0.09	4	0.22	0.01	4	0.09	0.05	4
B. Blood	2.73	0.36	4	1.52	0.30	4	0.89	0.04	4	0.35	0.08	4
C. Muscle	2.33	0.21	4	1.38	0.29	4	0.65	0.08	4	0.27	0.07	4
D. Spleen	2.99	0.55	4	1.59	0.45	4	0.81	0.02	4	0.27	0.05	4
E. Heart	4.22	0.67	4	2.10	0.45	4	0.95	0.10	4	0.33	0.08	4
F. Lung	3.98	0.57	4	2.16	0.15	4	0.79	0.06	4	0.43	0.07	4
G. Pancreas	5.11	0.65	4	2.83	0.36	4	1.19	0.11	4	0.44	0.17	4
H. Stomach	1.88	0.62	4	0.76	0.35	4	0.85	0.70	4	0.78	0.07	4
I. Small Intestir	13.50	7.96	4	18.94	11.11	4	14.37	4.11	4	4.05	0.41	4
J. Kidney	6.30	0.78	4	4.24	1.72	4	2.19	0.13	4	0.87	0.11	4
K. Liver	20.15	1.55	4	22.08	3.97	4	13.83	2.46	4	9.02	2.47	4
L. Bone	1.49	0.35	4	1.14	0.61	4	0.73	0.17	4	0.69	0.37	4

Mass calibration curves of compounds 1 and 2

The specific activity of compound **1** was calculated based on following mass calibration curve.

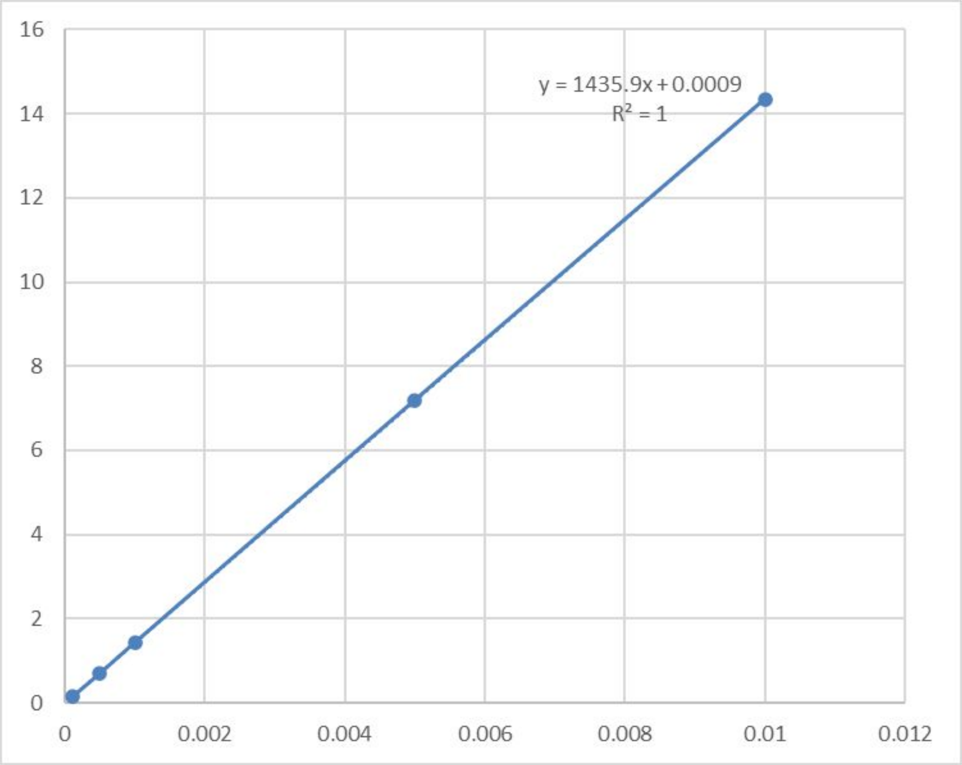

The specific activity of compound **2** was calculated based on following mass calibration curve.

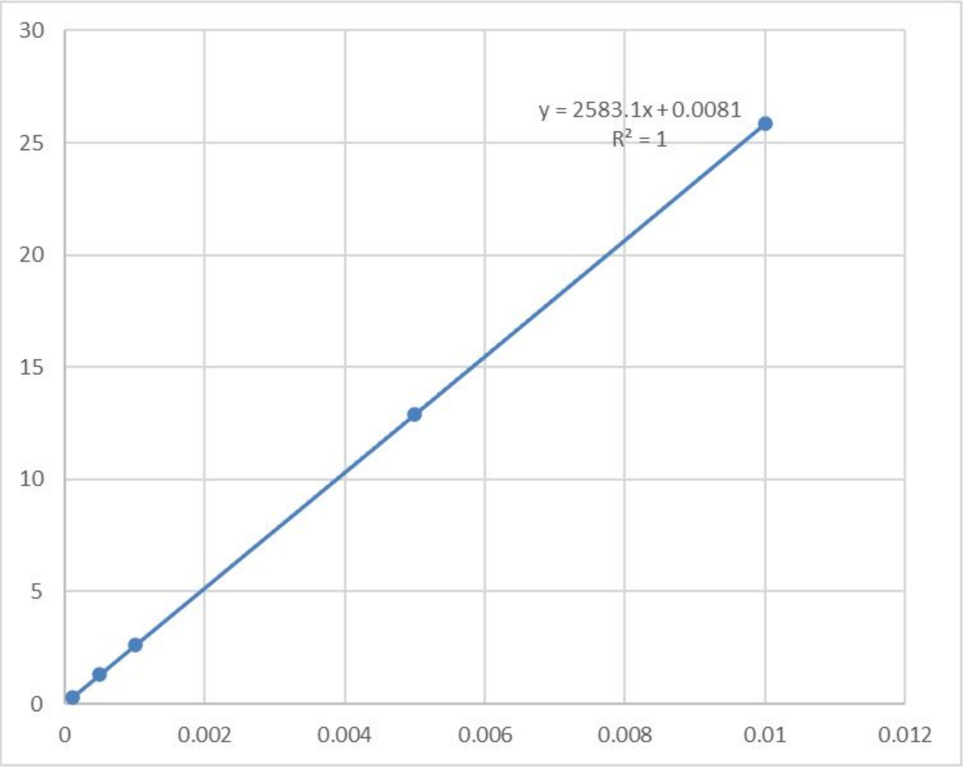

HPLC radio-chromatograms of [^{18}F]1 and [^{18}F]2

Semi-prep radio-HPLC chromatogram of [^{18}F]1

Column: Phenomenex Luna® 5 µm C18(2) 100 Å Prep Column (10 × 250 mm)

Mobile phase: CH_3CN-H_2O=50%-50% (containing 0.1% TEA)

flow rate: 5.0 mL/min

UV: 254 nm

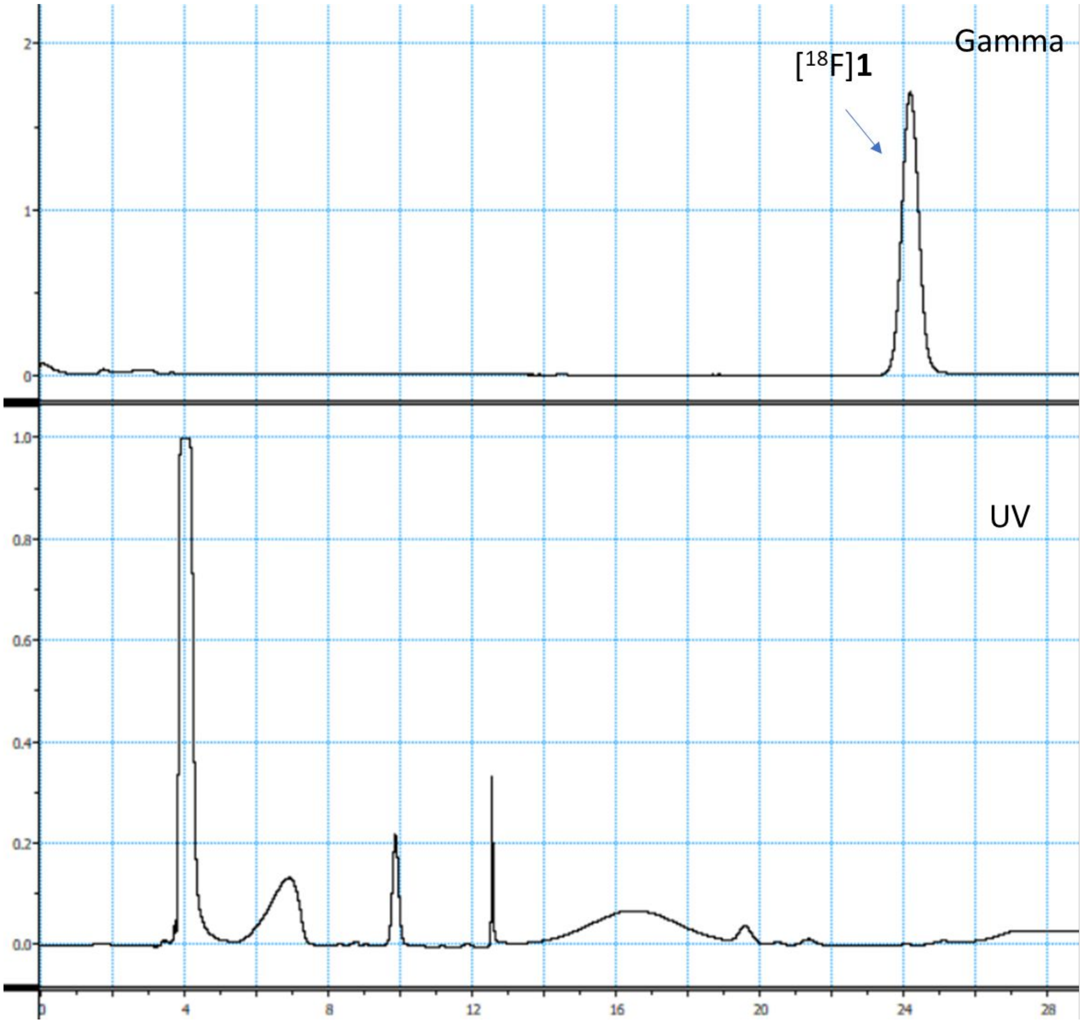

Analytical radio-HPLC chromatogram of [^{18}F]1

Column: XBridge C18 3.5 µm column (4.6 × 100 mm)

Mobile phase: CH_3CN-H_2O = 60%-40% (containing 0.1% TEA)

flow rate: 1.0 mL/min

UV: 254 nm

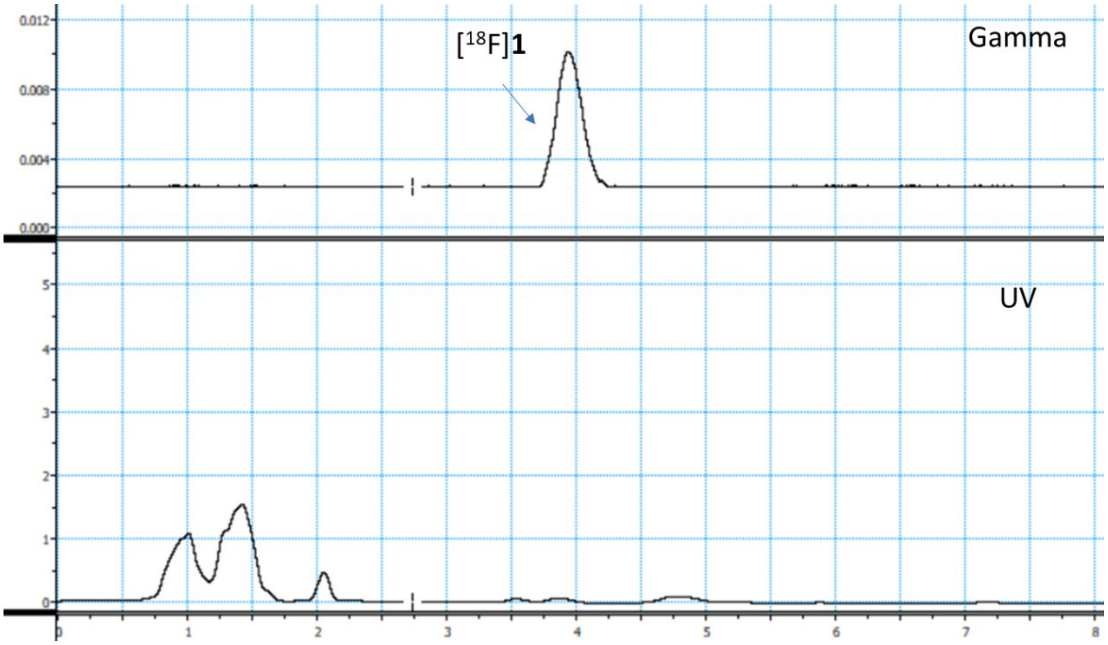

Co-injection of [^{18}F]1 with unlabeled 1

Column: XBridge C18 3.5 µm column (4.6 × 100 mm)

Mobile phase: CH_3CN-H_2O = 60%-40% (containing 0.1% TEA)

flow rate: 1.0 mL/min

UV: 254 nm

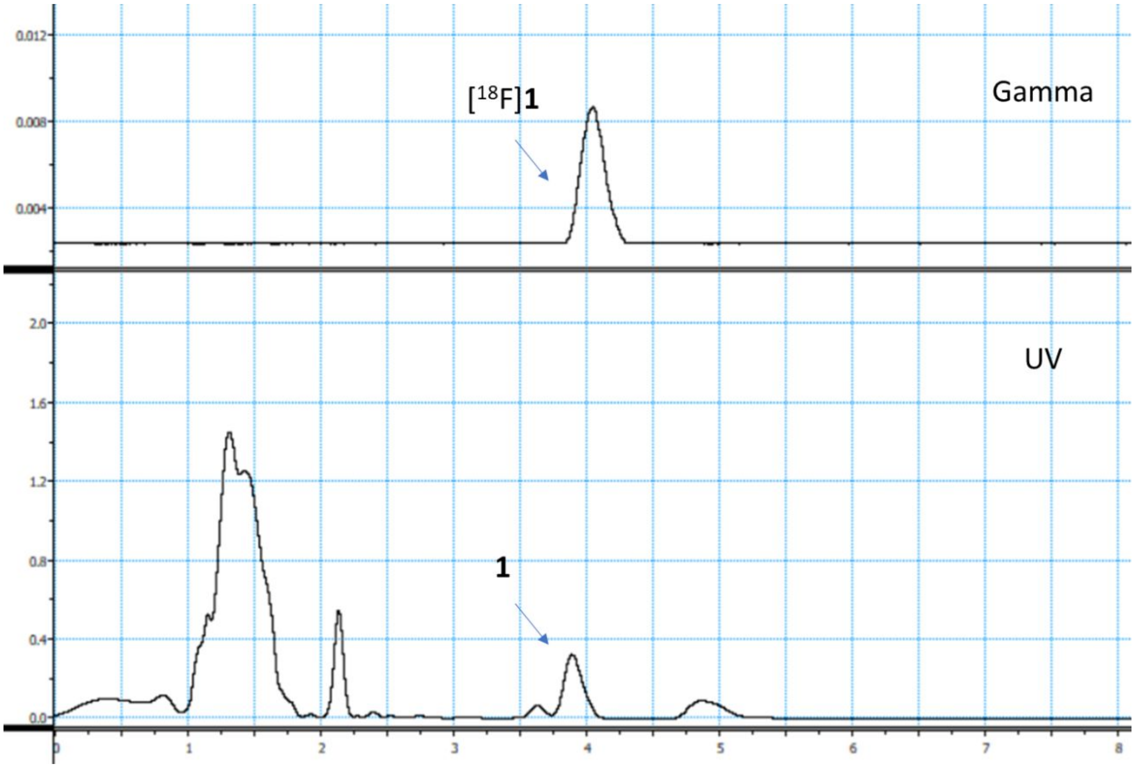

Semi-prep radio-HPLC chromatogram of [^{18}F]2

Column: Phenomenex Luna® 5 µm C18(2) 100 Å Prep Column (10 × 250 mm)

Mobile phase: CH_3CN-H_2O=50%-50% (containing 0.1% TEA)

flow rate: 5.0 mL/min

UV: 254 nm

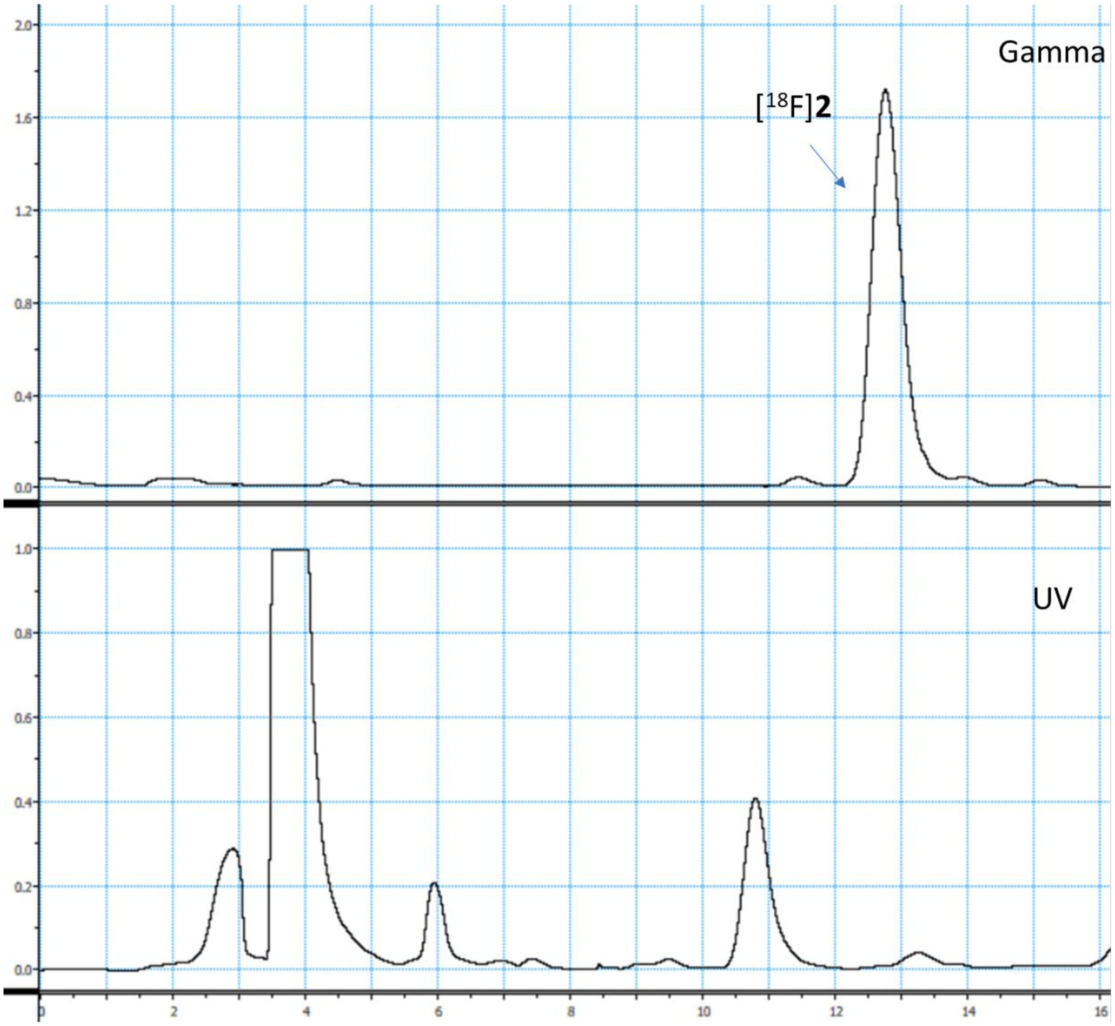

Analytical radio-HPLC chromatogram of [^{18}F]2

Column: XBridge C18 3.5 µm column (4.6 × 100 mm)

Mobile phase: CH_3CN-H_2O = 50%-50% (containing 0.1% TEA)

flow rate: 1.0 mL/min

UV: 254 nm

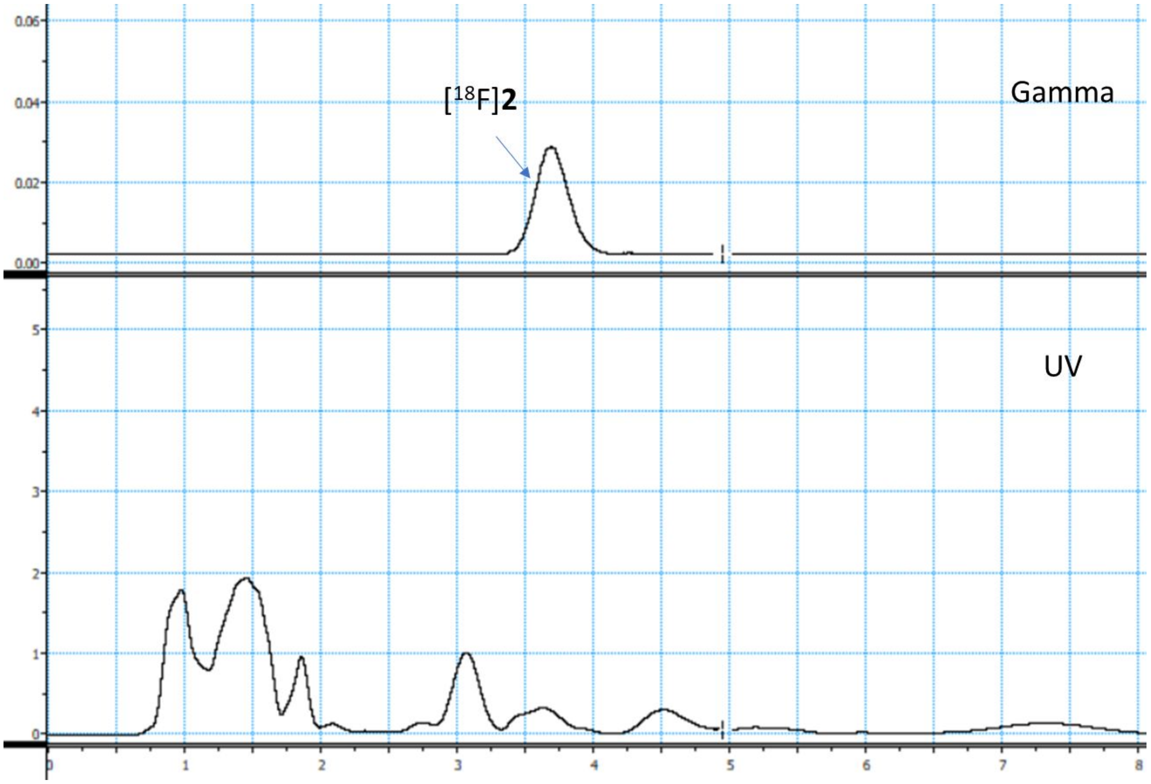

Co-injection of [^{18}F]2 with unlabeled 2

Column: XBridge C18 3.5 µm column (4.6 × 100 mm)

Mobile phase: CH_3CN-H_2O = 50%-50% (containing 0.1% TEA)

flow rate: 1.0 mL/min

UV: 254 nm

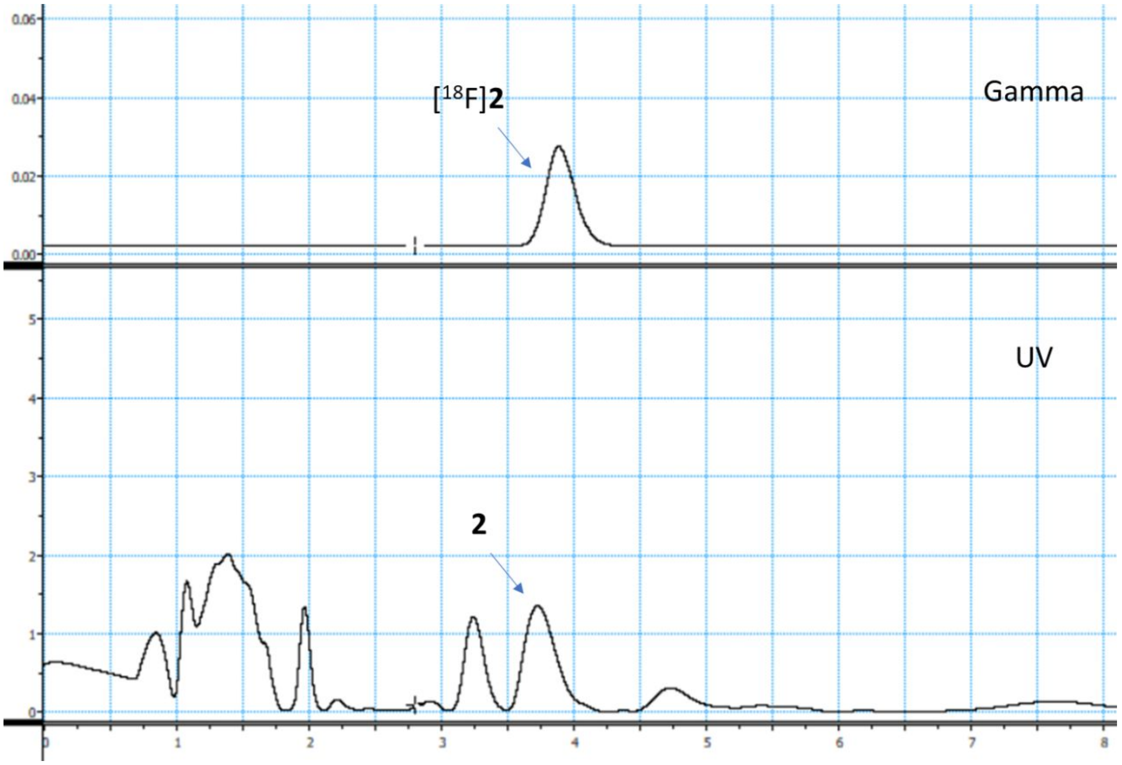

2. ^{1}H, ^{19}F, and ^{13}C NMR spectra of isolated compounds

^{1}H, ^{19}F, and ^{13}C NMR spectra of compound **1**

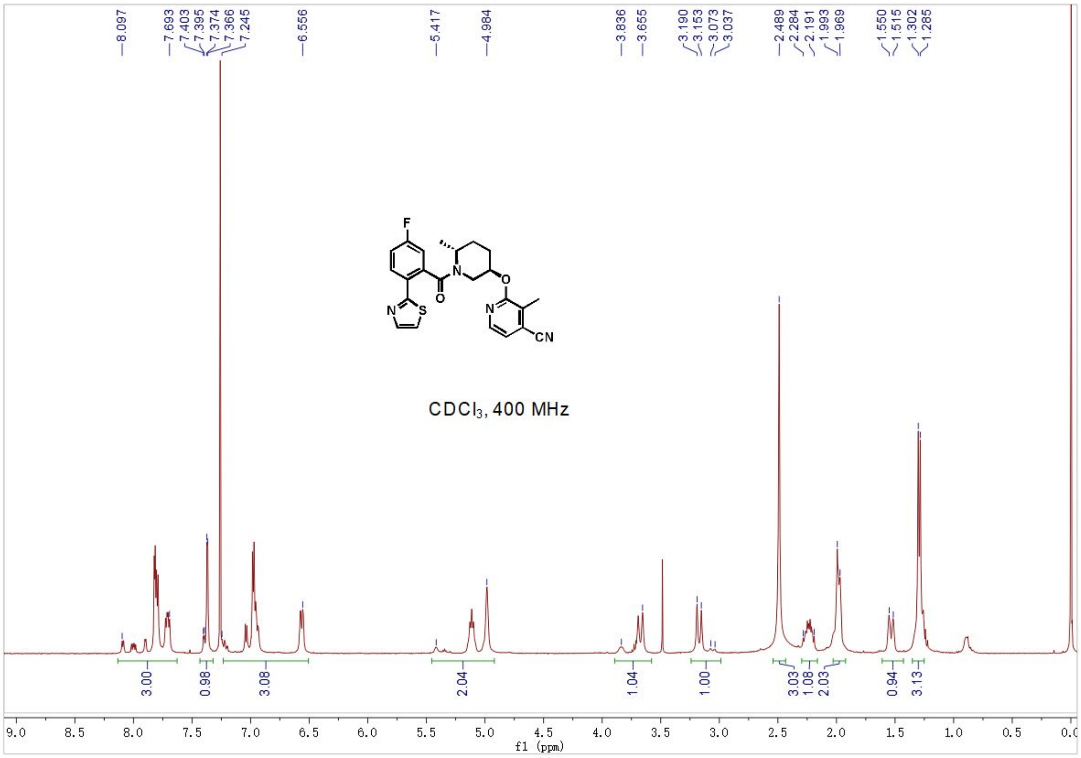

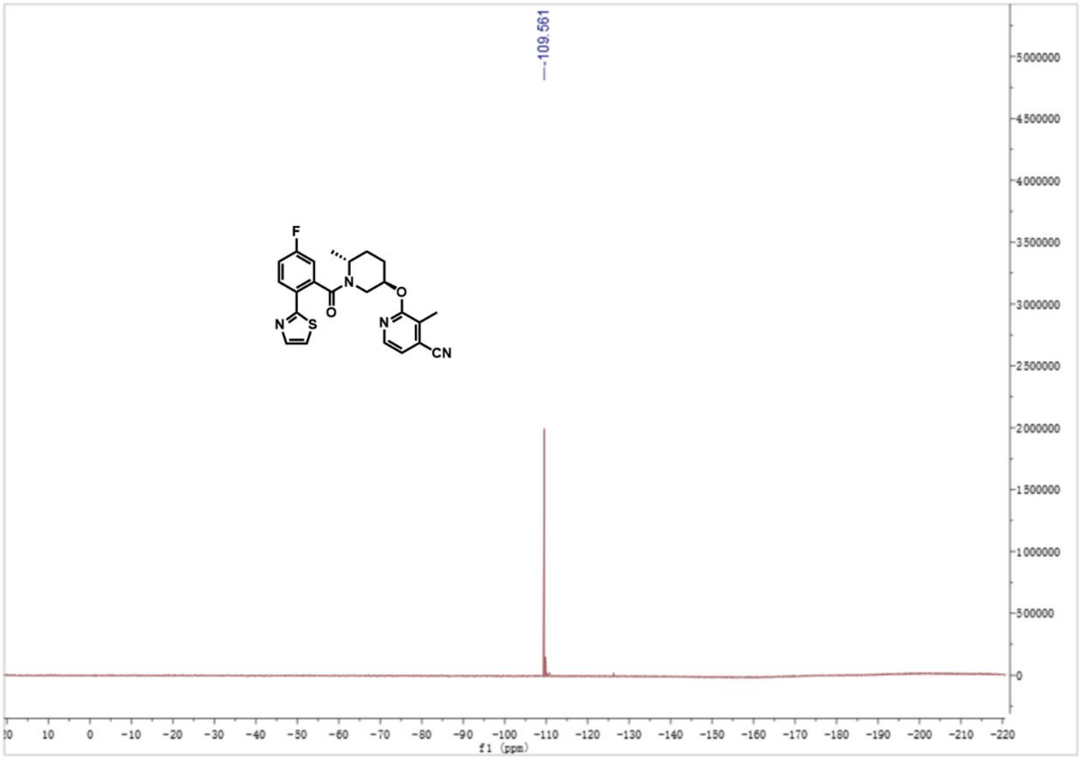

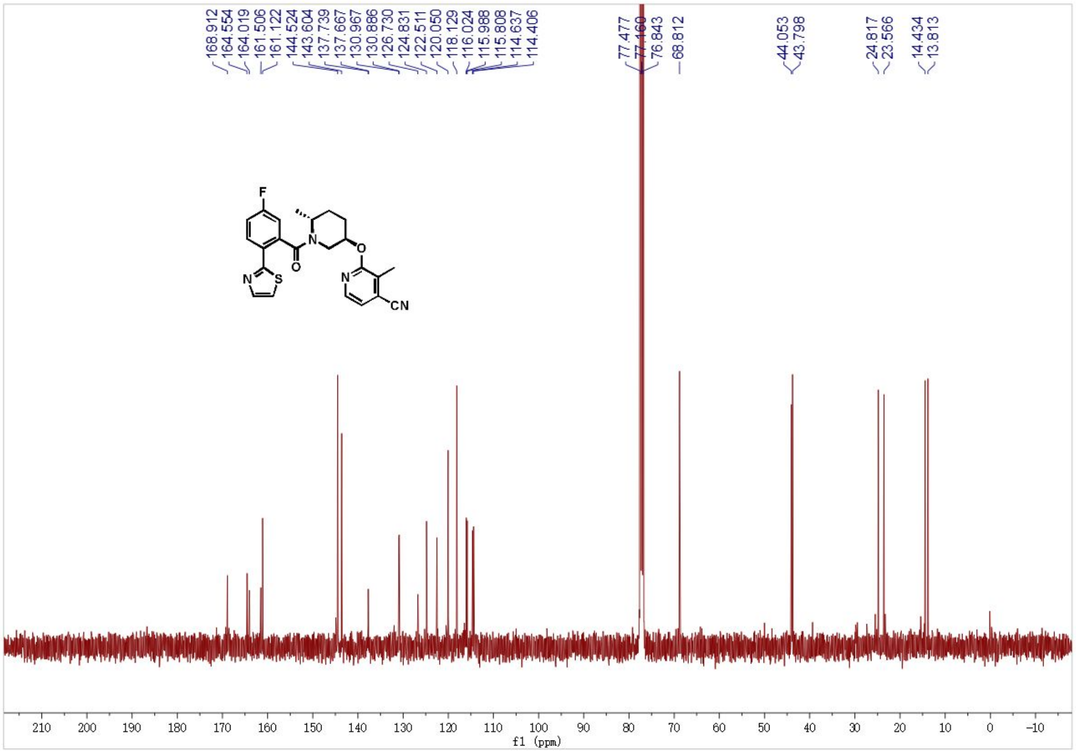

^{1}H, ^{19}F, and ^{13}C NMR spectra of compound **2**

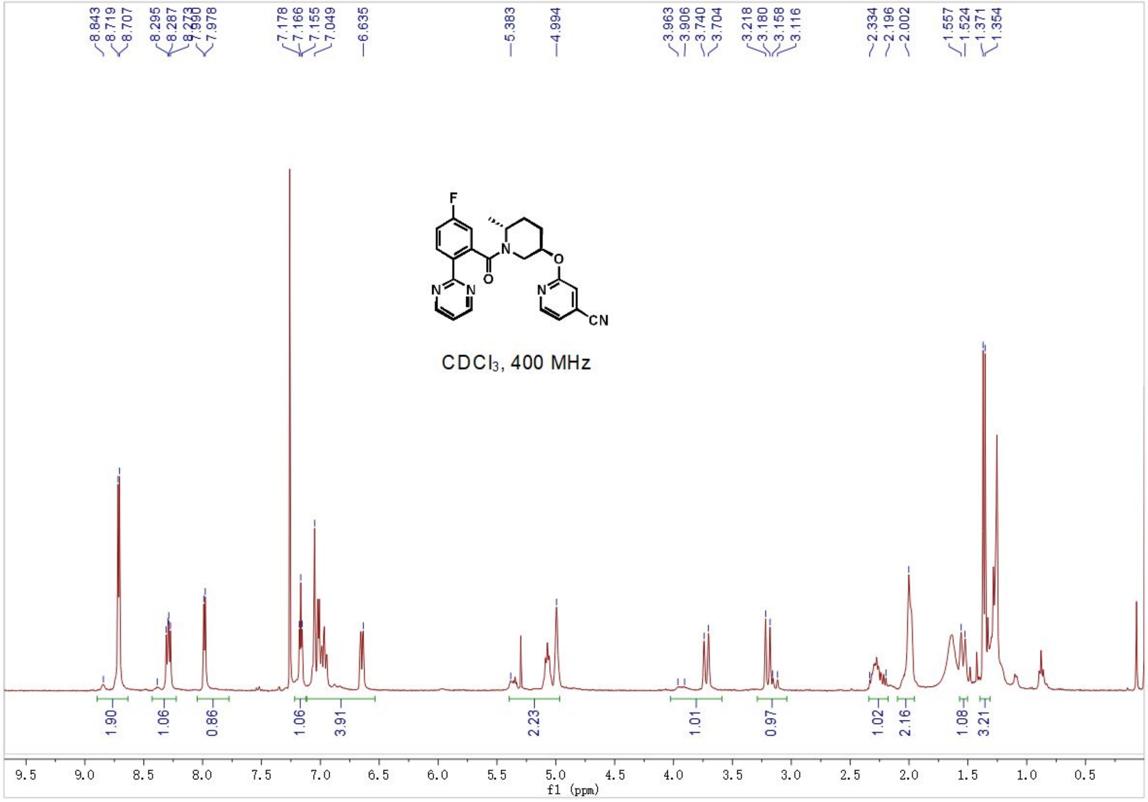

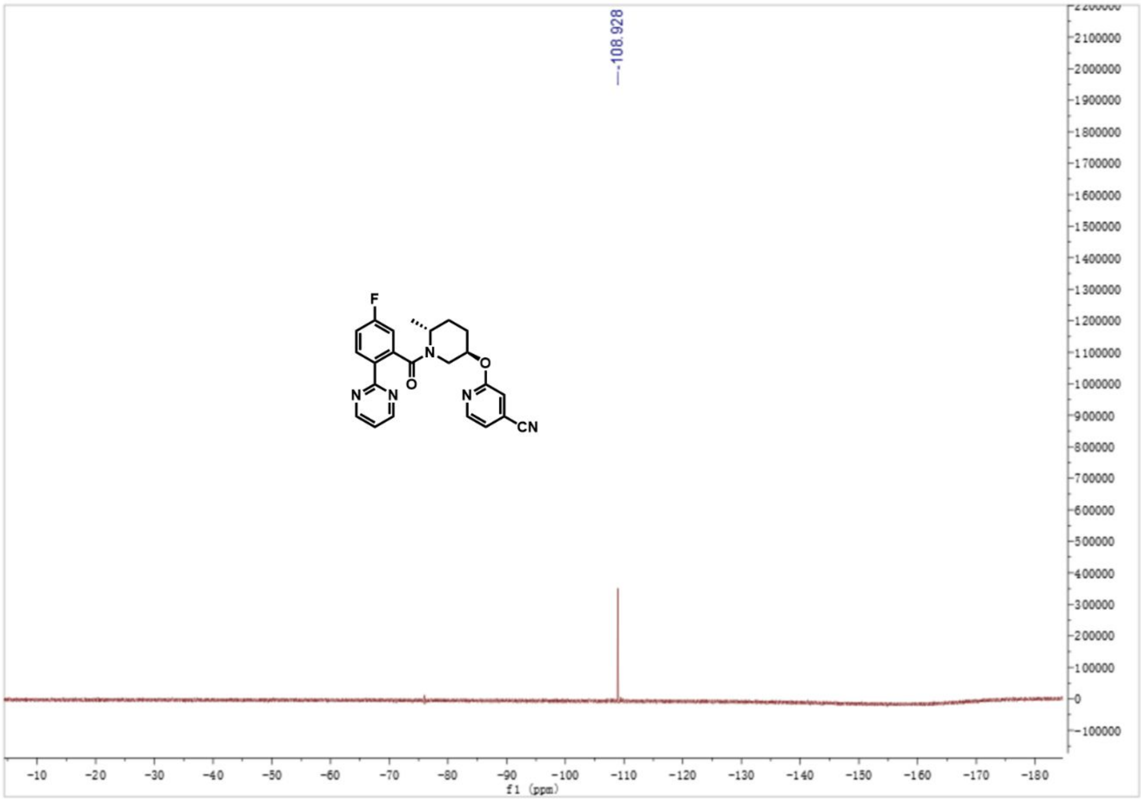
-108.928
F
N
O
CN
f1 (ppm)

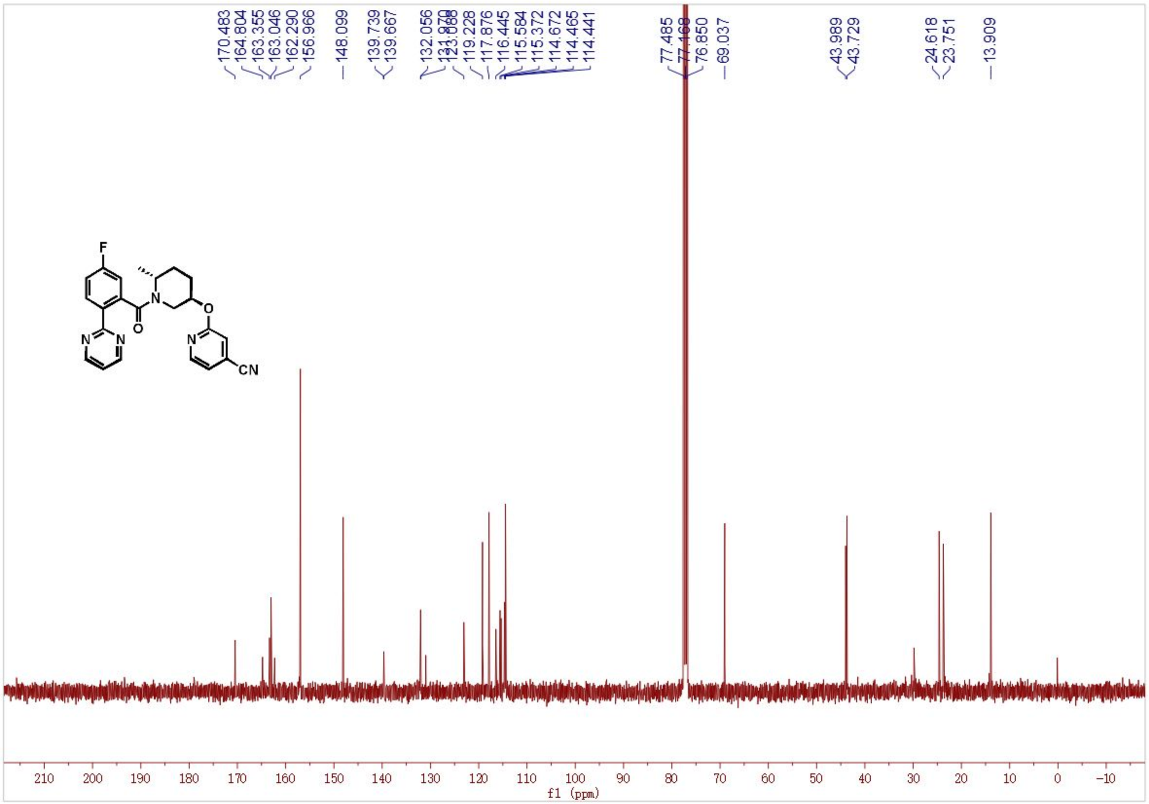
170.483
164.804
163.355
163.046
162.290
156.966
148.099
139.739
139.667
132.056
119.228
117.876
116.445
115.584
115.372
114.672
114.465
114.441
77.485
76.850
69.037
43.989
43.729
24.618
23.751
13.909
F
N
O
CN
f1 (ppm)

^{1}H and ^{13}C NMR spectra of compound **7**

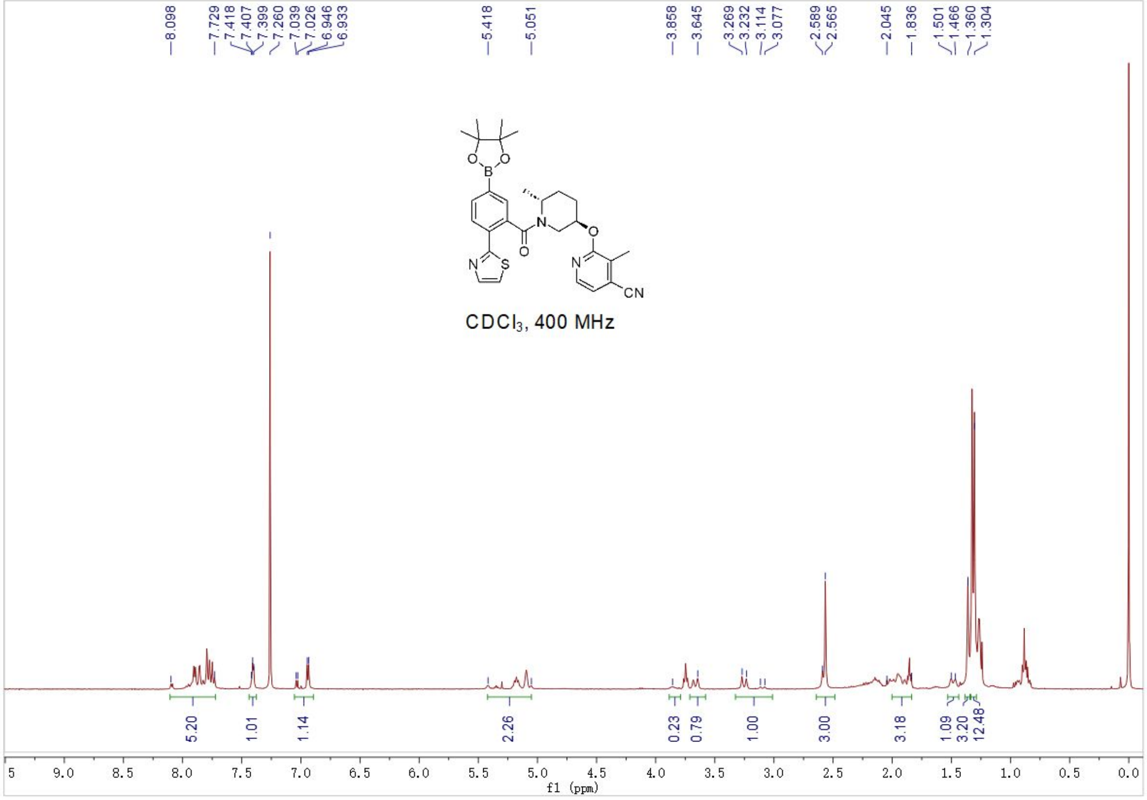

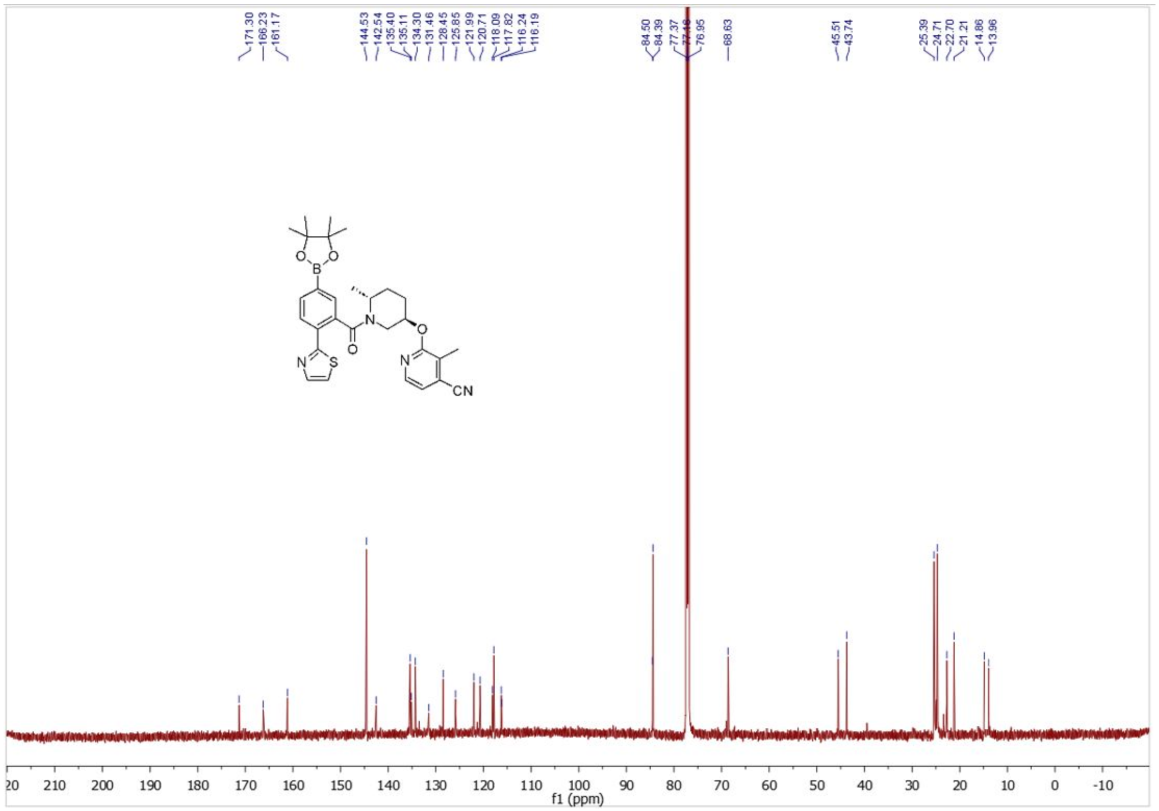

^{1}H and ^{13}C NMR spectra of compound **8**

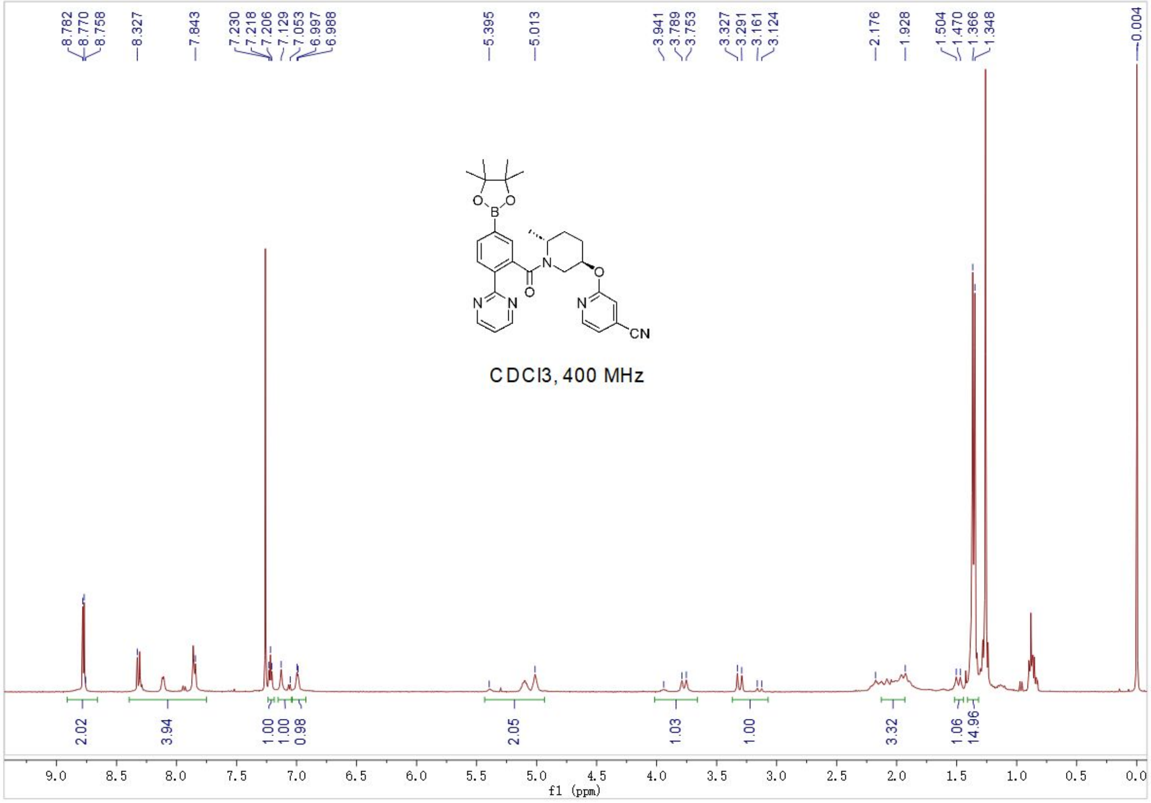

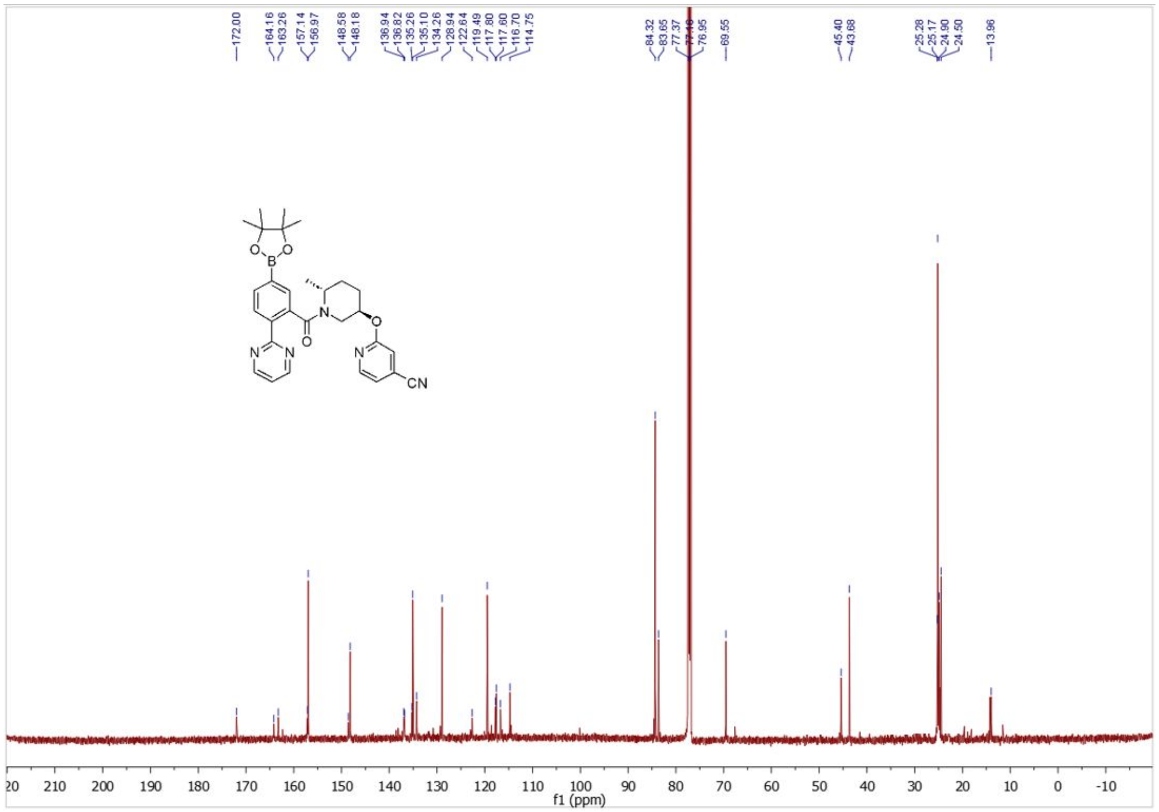

Supplement: Supplementary file 1 [file pt5c00474_si_001.pdf]
